# Reusable Pd-PolyHIPE for Suzuki–Miyaura Coupling

**DOI:** 10.1021/acsomega.1c06318

**Published:** 2022-04-06

**Authors:** Miha Ravbar, Amadeja Koler, Muzafera Paljevac, Peter Krajnc, Mitja Kolar, Jernej Iskra

**Affiliations:** † Faculty of Chemistry and Chemical Technology, University of Ljubljana, Večna Pot 113, 1000 Ljubljana, Slovenia; ‡ Faculty of Chemistry and Chemical Engineering, University of Maribor, Smetanova Ulica 17, 2000 Maribor, Slovenia

## Abstract

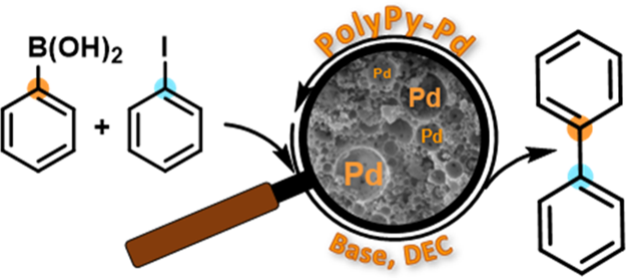

Palladium was immobilized on a highly porous copolymer of 4-vinylpyridine and divinylbenzene (polyHIPE—poly(high internal phase emulsion)) using palladium(II) acetate to obtain PolyPy-Pd with 6.1 wt % or 0.57 mmol Pd/g. The immobilized catalyst was able to catalyze the coupling of iodobenzene and phenylboronic acid in ethylene glycol monomethyl ether/water (3:1) within 4 h at rt and complete conversion was observed when 2.5 mol % of Pd per PhI was used. The reaction tolerated a wide range of substituents on the aromatic ring. Iodobenzene derivatives with electron-withdrawing substituents showed higher reactivity, while the opposite was true for the phenylboronic acid series. The polyHIPE-supported Pd catalyst was also used for the direct conversion of phenylboronic acid to biphenyl through an iodination/coupling reaction sequence. The recyclability of the heterogeneous catalyst was also optimized, and by finding a suitable combination of solvents for the loading of Pd, the reaction, and the isolation of the product, the solid-supported catalyst was completely regenerated and used in the next reaction with the same activity.

## Introduction

During the last half century, palladium-catalyzed reactions have become widely used in organic chemistry. Palladium catalysis enables transformations that are not readily possible with classical techniques. In most cases, the reactions proceed under mild reaction conditions and tolerate a wide range of different substrates. Palladium-catalyzed C–C coupling reactions have been the target of a variety of different studies since their introduction in the 1970s.^1^

In the Suzuki–Miyaura reaction, organic electrophiles such as aryl, alkenyl, or alkynyl halides and triflates are coupled with organoboron compounds in the presence of a base.^2^ The first example was published in 1979, and interest in the reaction increased steadily thereafter.^3−5^ The impact of the Suzuki–Miyaura reaction on organic synthesis has been quite significant, enabling the synthesis of building blocks for many polymers, ligands, and a large number of natural products and biologically active pharmaceutical compounds.^6−9^ The Suzuki reaction has many advantages, including mild reaction conditions, high tolerance to functional groups, commercial availability of organoboron compounds, and their stability in water and air. The by-products formed in the reaction are nontoxic and can be easily separated from the reaction mixture. However, the reaction is still usually catalyzed by palladium complexes and salts with added ligands. This means that the palladium is lost during the reaction, which reduces the economics of the process. Moreover, for biologically active substances and pharmaceuticals, the palladium must be removed from the reaction mixture.

However, the problems can be mitigated or solved by the use of heterogeneous palladium catalysts, which allow easy separation from the reaction mixture and subsequent reuse.^10^ One of the most commonly used solid supports for palladium in Suzuki–Miyaura catalysis is activated carbon (Pd/C).^11−13^ Metal oxides have also been used successfully. For example, KF/Al_2_O_3_ doped with palladium, perovskites, and iron oxide coated with a polymer layer containing an immobilized Pd–N-heterocyclic carbene complex.^14−16^ Various zeolites and silica have also been used.^17−20^ A number of other inorganic materials that have been successfully used as supports for palladium include sepiolite clay [Mg_8_Si_12_O_30_(OH)_4_·4H_2_O·*n*H_2_O], Ca-deficient hydroxyapatite [Ca_9_(HPO_4_) (PO_4_)_5_(OH)], and Mg–Al-layered double hydroxide [Mg_0.75_Al_0.25_(OH)_2_(Cl)_0.25_·*z*H_2_O].^21−23^ On the other hand, polymers are becoming increasingly popular as supports for metal catalysts. Because of their versatile physical properties and chemical functionality, they can be specially designed to provide solid support with the right properties. For example, chloromethylated polystyrene resin was treated with LiPPh_2_ to obtain a phosphinated resin on which Pd was then immobilized.^24^ This heterogeneous catalyst proved effective for coupling a variety of organoboranes with 1-alkenyl bromides or iodobenzene and triflates. The catalyst could also be recycled 5–10 times without a decrease in activity. In another study, a porous polydivinylbenzene functionalized with a Shiff base was used as a solid support on which palladium was immobilized by palladium acetate.^25^ The catalyst proved to be very efficient in catalyzing the Suzuki coupling of phenylboronic acid with aryl iodides and aryl bromides (95–99% yield within 20 min at 80 °C), even at a very low catalyst loading of 0.1–0.01 mol %. The most interesting polymeric support for Pd is the one with embedded pyridine, either as a mesoporous composite of a linear poly(4-vinylpyridine) and tetrachloropalladate^26^ or as poly(styrene-*co*-4-vinylpyridine) microspheres with embedded Pd nanoparticles.^27^

High internal phase emulsions (HIPEs) have been known for some time and are used in many fields, from food preparation to cosmetics.^28,29^ Their defining feature is an internal phase that accounts for at least 74% of the volume of the emulsions. This value corresponds to the theoretical volume fraction of non-deformed, efficiently stacked spheres. HIPEs can have an even higher volume fraction of the internal phase, even up to 99%. Polymers synthesized with HIPEs are termed polyHIPEs. Under the right conditions, small pores form between the droplets of the internal phase, allowing the internal phase to be removed by drying. This creates a very porous material with cellular interconnected porosity.^28^ Because of their open porosity, polyHIPEs are useful as solid supports for reaction components. An example of the use of a polyHIPE polymer [poly(4-vinylbenzyl chloride-*co*-divinylbenzene)-triethylenediamine motif] is the synthesis of the first renewable polymer reagent for electrophilic fluorination.^30^ The efficient fluorination reagent was a result of the high material porosity, which allowed the transfer of substrates to the fluorine atom immobilized on the polymer. PolyHIPE based on 4-vinylpyridine has been successfully used as a solid support for copper in copper-catalyzed azomethine-imine-alkyne cycloadditions (CuAIAC).^31^ Other uses of polyHIPE are also possible, such as a material for plutonium separation, where 4-vinylpyridine was grafted onto polyHIPE monoliths to increase the anion-exchange capacity of the material.^32^ The Suzuki–Miyaura reaction was already used for the modification of polyHIPE.^33^

In this report, we describe the use and potential of poly(4-vinylpyridine-*co*-divinylbenzene)-polyHIPE as a solid support for palladium in the catalysis of the Suzuki–Miyaura reaction between arylboronic acids and aryl iodides.

## Results and Discussion

**Figure 1:**
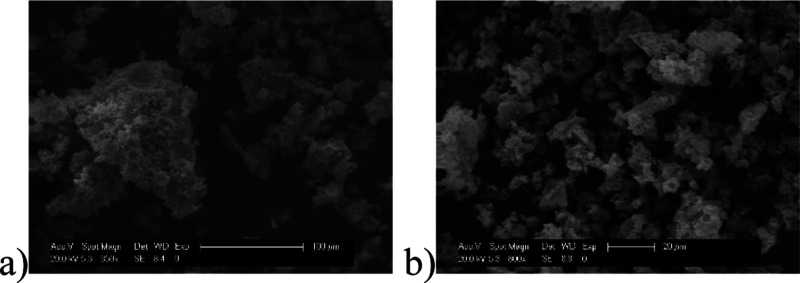
(a) PolyHIPE polymer—polyPy and (b) polyHIPE polymer with immobilized palladium—polyPy-Pd.

Palladium was immobilized on the polyPy with a solution of Pd(OAc)_2_ in MeCN. The resulting heterogeneous catalyst polyPy-Pd was characterized by scanning electron microscopy (SEM) and by determining the amount of palladium immobilized on the polyHIPE support. The SEM images showed no deformation of the morphology of the support during the immobilization of palladium (Figure 1). An open-pore, cellular morphology with primary pores approximately 10 μm in diameter and numerous interconnected channels can be seen. There was no evidence of occluded salt in the polymer cavities. The hypercross-linking of the supporting polyHIPE polymer enabled several advantages over a plain polymer support. Namely, the increased density of cross-links in parts of the polymer (hypercross-linked domains) caused the induction of mesopores as suggested by the increased surface area compared to a non-hypercross-linked polyHIPE support (76 m^2^/g compared to 16 m^2^/g). The mechanical stability of the supporting polymer was also improved by the cross-linking, enabling easier handling of the solid-supported catalyst. The amount of palladium immobilized on the polymer support was determined after sample digestion using atomic adsorption spectroscopy (AAS). The palladium loading was 6.1 wt %, or 0.57 mmol/g. ethylene glycol monomethyl ether (EGME) was not suitable as a solvent for catalyst loading as polyPy-Pd turned black.

PolyPy-Pd was tested as a catalyst in the Suzuki–Miyaura reaction with the model substrates iodobenzene **1a** and phenylboronic acid **2a**. First, the effect of different solvents on this reaction was tested (Table 1). Water was used in combination with each of the solvents at a ratio of 3:1 (solvent/water), as it was previously found that this accelerated the reaction.^34^ 2.5 mol % of the Pd catalyst in the form of polyPy-Pd relative to **1a** was used. The reaction proceeded best in EGME, where complete conversion was achieved within 4 h. The conversion was slightly lower in the less polar solvents dichloromethane (DCM) and toluene with 95–97% conversion after 4 h and even lower in alcohols with 78% conversion in ethanol and 86% in methanol after 4 h. Acetonitrile, dimethoxyethane, and propylene carbonate were significantly less effective, with conversions below 62% at 4 h reaction time. Because the reaction in EGME was the most efficient, it was used in further experiments.

**Table 1:**
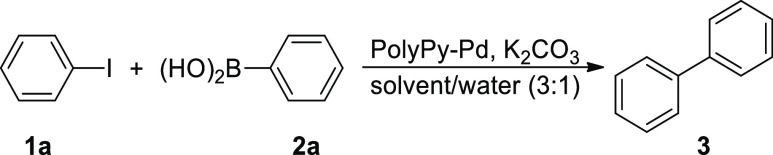
Effect of Solvent on the Suzuki–Miyaura Reaction between Iodobenzene (**1a**) and Phenylboronic Acid (**2a**) Catalyzed by polyPy-Pd^a^

	conversion^b^		
solvent	10 min (%)	1 h (%)	4 h (%)
EGME	93	96	≥99
MeOH	87	84	86
EtOH		79	78
DME		70	62
MeCN		32	62
toluene		90	95
DCM		98	97
propylene carbonate		33	33

a 0.5 mmol **1a**, 0.6 mmol **2a**, 0.6 mmol K_2_CO_3_, 22 mg polyPy-Pd (2.52 mol % based on **1a**), and 2 mL of solvent/water (3:1).

b Conversion is determined from the ratio of ^1^H NMR signals **1a**/**3**.

Next, we reduced the amount of polyPy-Pd and compared its reactivity with that of Pd(OAc)_2_ (Table 2). Reducing the amount of the catalyst led to slower reactions, and reactions were not completed after 4 h of reaction time. When palladium acetate was used, the reaction with 0.63 mol % Pd was slower than when 2.52 mol % Pd of polyPy-Pd was used, but the conversion was quantitative after 4 h of reaction.

**Table 2:**
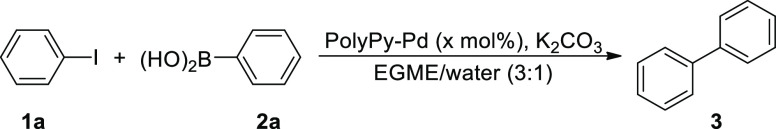
Effect of the Amount of the Catalyst on the Suzuki–Miyaura Reaction between **1a** and **2a**^a^

	conversion^b^		
catalyst (mol %)	10 min (%)	1 h (%)	4 h (%)
2.52%	93	96	≥99
1.26%	86	89	93
0.63%	80	84	88
0.63%^c^	87	92	≥99

a 0.5 mmol **1a**, 0.6 mmol **2a**, 0.6 mmol K_2_CO_3_, *x* mol % polyPy-Pd (based on **1a**), and 2 mL of solvent.

b Conversion is determined from the ratio of ^1^H NMR signals **1a**/**3**.

c Pd(OAc)_2_ as a catalyst.

We further tested the extent of the Suzuki–Miyaura reaction using polyPy-Pd as the catalyst. First, different iodobenzenes **1** were tested by coupling with **2a** under previously optimized conditions (EGME/water as the solvent and 2.52 mol % Pd, 24 h) (Table 3). The reactivity of iodobenzenes **1** with electron-donor substituents was first tested. 4-Methoxyiodobenzene **1b** was slightly less reactive than **1a**, with 92% conversion after 4 h, while complete conversion was achieved in 24 h. 3-Iodoaniline **1c** was even less reactive, with 82% conversion after 24 h. The use of 3-methyliodobenzene **1d** showed no change in reactivity compared to **1a**, but the 4-*tert*-butyl derivative **1e** was less reactive and product **7** could not be separated from **1e**. Iodobenzenes with electron-withdrawing groups were more reactive, with 4-chloro-**1f**, 3-chloro-**1g**, and 3-nitroiodobenzene **1h** achieving complete conversion within 1 h, except for pentafluoroiodobenzene **1i**. Iodobenzene with acidic 4-carboxyl group **1j** was converted only 79% after 4 h and did not increase with longer reaction time.

**Table 3:**
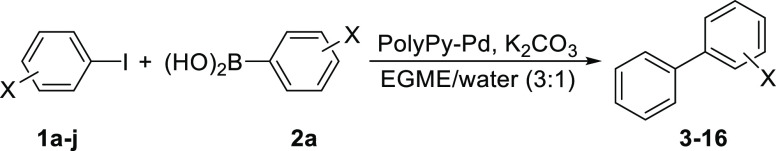
Suzuki–Miyaura Coupling of Iodobenzenes and Phenylboronic Acids^a^

X-PhI	X-PhB(OH)_2_	conversion^b^ (%)	yield^c^ (%)
H (**1a**)	H (**2a**)	≥99	3:92
4-OMe (**1b**)	H (**2a**)	≥99	4:88
3-NH_2_ (**1c**)	H (**2a**)	82	5:57
3-Me (**1d**)	H (**2a**)	≥99	6:93
4-tBu (**1e**)	H (**2a**)	90	7^d^:/
4-Cl (**1f**)	H (**2a**)	≥99	8:87
3-Cl (**1g**)	H (**2a**)	≥99	9:83
3-NO_2_ (**1h**)	H (**2a**)	≥99	10:81
F_5_-(**1i**)	H (**2a**)	98	11:66
4-COOH (**1j**)	H (**2a**)	79	12:55
H (**1a**)	4-tBu (**2b**)	≥99	7:87
H (**1a**)	4-(4′-OMePhCH_2_O) (**2c**)	≥99	13:75
H (**1a**)	4-CN (**2d**)	≥99	14:88
H (**1a**)	4-NO_2_ (**2e**)	90	15:66

a 0.5 mmol **1**, 0.6 mmol **2a**, 0.6 mmol K_2_CO_3_, 22 mg polyPy-Pd (2.52 mol % based on **1**), and 2 mL of EGME/water (3:1).

b Conversion after 24 h of reaction was determined by ^1^H NMR.

c Yield is determined based on the mass of pure, isolated product.

d Product **7** could not be separated from starting **1e** by column chromatography and was isolated as a mixture with **1e**.

Various substituted phenylboronic acids were also tested. Phenylboronic acids **2** containing electron-donor substituents (**2b** and **2c**) had similar reactivity to **2a**. Phenylboronic acid with electron-withdrawing groups (**2d** and **2e**) was less reactive and, after 4 h, **2d** had an 83% conversion and **2 × 10**^**91**^%. **2d** was completely converted to **14** after 24 h, while the yield of **15** did not increase further even after 24 h. The reactivity is in accordance with the general rules for the Suzuki–Miyaura reaction. Despite the use of a heterogeneous catalyst, tolerance to a variety of different functional groups on substrates is maintained, and the formation of by-products was not observed.

**Figure 2:**
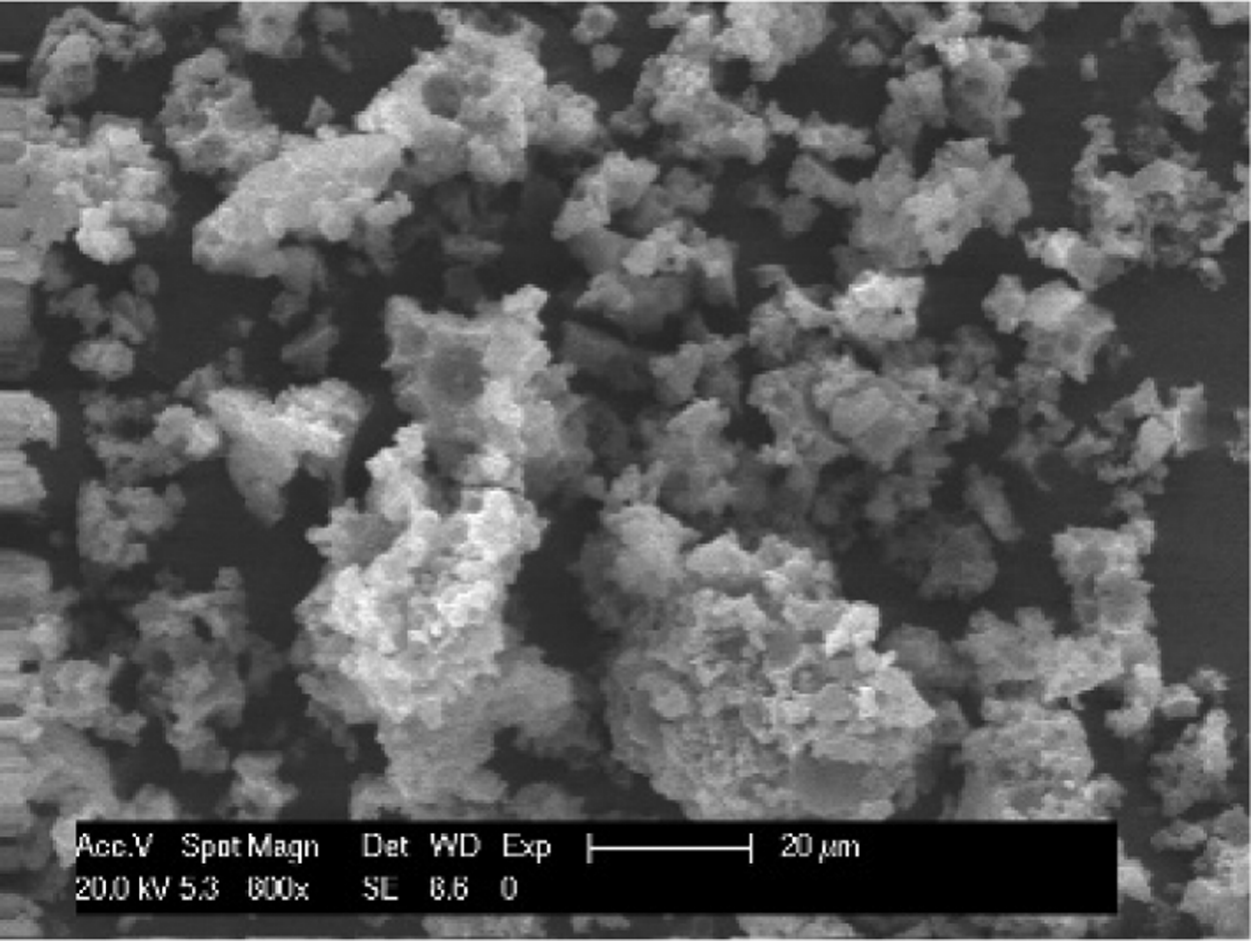
Heterogeneous catalyst after first reaction.

The reuse of polyPy-Pd was studied in the reactions of **1a** and **2a** under the same conditions as in the preparative reactions. After the reaction, polyPy-Pd was separated by filtration, washed with EGME, and air dried. The regenerated polymer had a higher mass due to the presence of salts, so the catalyst was washed with methanol, filtered off, and air-dried. The catalyst was then reused in a subsequent reaction using the same procedure. The whole process was repeated for a total of three subsequent reactions using the same catalyst (Table 4, Procedure A). The activity of the catalyst decreased after reuse, with 79% conversion after 4 h and 94% after 24 h. However, the decrease in catalyst activity is lower between the second and third cycles. Thus, most of the loss of catalyst activity occurs during the first reaction, which is in line with the decrease of the amount of Pd on the solid support. The SEM images show no change in the morphology of the polymer and no occluded salts (Figure 2). This confirms the leaching of palladium as the cause of the loss of catalyst activity. We tested whether the reaction is catalyzed by the soluble Pd or the one on the solid support. PolyPy-Pd was stirred in EGME/water 3:1 for 24 h and then removed by filtration. PolyPy-Pd was filtered out and the same reaction as mentioned above was carried out with the polymer and with the mother liquor. In both cases, complete conversion was achieved. We replaced the lost palladium between the follow-up reactions by adding polyPy-Pd into an acetonitrile solution of palladium acetate for 24 h, followed by washing and air-drying (Table 4, Procedure B). The amount of Pd on the polyPy-Pd was recovered, as was the reactivity for three successive reactions. Next, Pd leaching during the reaction in various suitable solvents was tested (Table 1). The reactions were carried out according to the same procedure as described above, except that the solvent was either toluene or DCM. In both cases, complete conversion was achieved in 4 h (Table 4, Procedure C). The leaching of Pd was lower, but the color of the catalyst changed from light orange-brown to dark grey or even black, indicating an undesirable formation of palladium black.^35^ We also investigated whether we could reduce the leaching of palladium by changing the work-up procedure. Washing the catalyst after the reaction with acetonitrile instead of methanol led to an even lower amount of Pd on polyPy-Pd (acetonitrile: 30%; methanol: 41%—relative to the amount before the reaction). Finally, we tested whether we could redeposit leached palladium on the polymer directly after the reaction by adding DCM to the reaction mixture. After the filtration and drying of the polyPy-Pd, the content of Pd was 70% of the starting amount. The recovered catalyst was directly reused with the same efficiency (Table 4, Procedure D).

**Table 4:** Reuse of the Catalyst for Suzuki Coupling of **1a** and **2a**

		conversion^a^			
Procedure^b^	run	1 h (%)	4 h (%)	24 h (%)	% Pd^c^
A	1	96	≥99	≥99	41%^d^
	2	60	79	94	36%
	3	50	79	90	30%
B	1	96	≥99		85%^d^
	2	92	96		
	3	96	≥99		
C	PhCH_3_		≥99		62%^d^
	DCM		≥99		87%^d^
D	1	96	≥99		70%^d^
	2	≥99			
E	1	86	93	≥99	100%^e^
	2	91	≥99		

a Reaction conditions are as in Table 3. Conversion is determined from the ratio of ^1^H NMR signals **1a**/**3**.

b Procedure A: polyPy-Pd was filtered off, washed with EGME and MeOH, and dried. Procedure B: polyPy-Pd was put into the acetonitrile solution of Pd(OAc)_2_ for 24 h before reusing. Procedure C: EGME was replaced by PhCH_3_ or DCM. Procedure D: after the reaction, DCM was added and polyPy-Pd filtered off and reused directly. Procedure E: prior to the reaction, polyPy-Pd was washed by EGME. After the reaction, DEC was added and polyPy-Pd filtered off and reused directly.

c A % of Pd on the polymer support after the reaction (relative to the starting amount of Pd) is determined by AAS after the digestion of a weighted amount of the catalyst.

d Pd content before the reaction was 6.1 wt %.

e Pd content before the reaction was 3.0 wt %.

Because of the increasing concern regarding the use of chlorinated solvents, a possible better alternative was considered. The best results were achieved using diethyl carbonate (DEC). To further minimize leeching during the reaction, the prepared polyPy-Pd was washed with the reaction solvent (EGME) before the reaction, and the palladium content decreased from 4.6 to 3.0 wt %. This, in combination with the addition of DEC after the reaction to redeposit leached Pd to polyPy, essentially eliminated the leaching of palladium during the reaction, and the catalyst could be reused without any loss of effectiveness (Table 4, Procedure E).

**Scheme 1:**
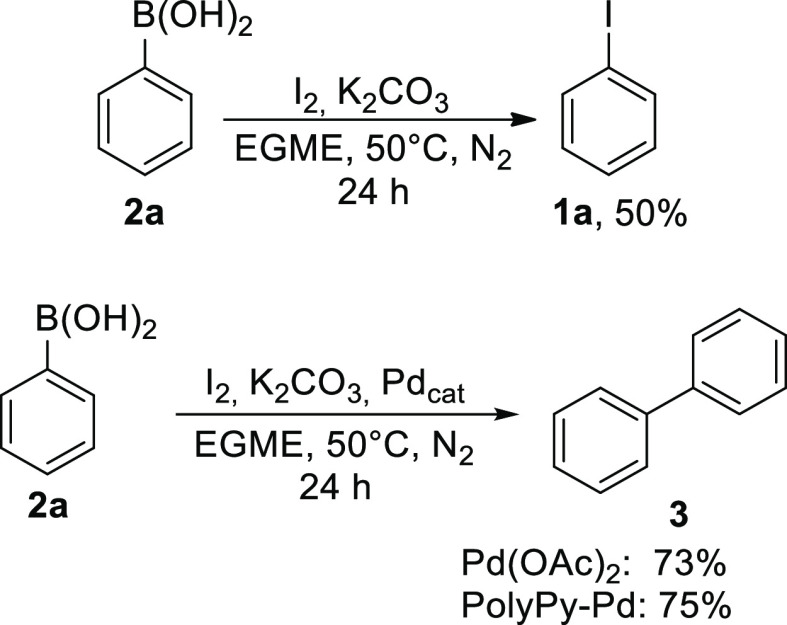
One-step Iodination and Cross-Coupling Reaction of Phenylboronic Acid

In the final part of this study, we investigated the possibility of synthesizing biphenyl **3** from **2a** alone. First, we tested the literature method for the iodination of **2a** with iodine (Scheme 1).^36^ Despite a lower yield (50%), pure **1a** was obtained without further purification because the residual **2a** was removed during extraction by the addition of the base. Next, the iodination of **2a** and the subsequent Suzuki–Miyaura reaction were carried out in one step. The reaction was set up as before, using only 0.5 equivalents of iodine and adding the palladium catalyst. Both our heterogeneous catalyst and palladium acetate were tested and similar yields (75 and 73%, respectively) (Scheme 1) were obtained in both cases. The cross-coupling procedure is very simple and requires only phenylboronic acid, while the entire biphenyl synthesis can be carried out in a one-step reaction.

## Experimental Section

### Materials and Methods

Starting compounds and solvents were obtained from commercial sources. 37% hydrochloric acid (Honeywell), 40% hydrogen peroxide, sodium sulphite (Merck), and Pd standard of 1000 μg/mL (J.T. Baker)

Poly(pyridine-*co*-divinylbenzene) was prepared using a procedure from the literature.^31^ The amount of pyridine rings in the polymer was determined by elemental analysis to be a 1,1 mmol/g polymer. The surface area of the supporting polymer was measured using the nitrogen adsorption/desorption method on a Micromeritics Tristar II (Micromeritics Inc., USA) porosimeter and was found to be 76 m^2^/g.

NMR spectra were recorded using a BRUKER DPX 300 NMR spectrometer and a BRUKER AVANCE III 500 MHz NMR spectrometer. Chemical shifts are given in reference to tetramethylsilane (^1^H: δ = 0 ppm), CHCl_3_ (^1^H: δ = 7.26 ppm, ^13^C: δ = 77.2 ppm), or dimethyl sulfoxide (DMSO) (^1^H: δ = 2.5 ppm, ^13^C: δ = 39.5 ppm) for ^1^H or ^13^C spectra, and CFCl_3_ (^19^F: δ = 0 ppm) for ^19^F spectra.

The amount of palladium on the polymer was determined using a Varian AA240 atomic absorption spectrometer (λ = 247.6 nm, Δλ = 0.2 nm, Φ_(air)_ = 3.5 L/min, Φ_(C_2_H_2_)_ = 1.5 L/min, and *i*_(hollow cathode)_ = 5 mA).

### Preparation of the Catalyst

A round-bottom flask was charged with 557 mg of palladium(II) acetate and then dissolved in 20 mL of acetonitrile. 600 mg of the polymer was added. The mixture of the polymer and palladium acetate solution was stirred on a magnetic stirrer for 24 h at room temperature. The polymer was filtered off and washed with acetonitrile. The color of the polymer changed from white to light orange during the reaction. The polymer was air dried, yielding 634 mg of dry polyPy-Pd containing 6.1 wt % Pd, which was used as a catalyst.

### General Procedure for the Optimization Reactions

A round-bottom flask was charged with 102 mg of iodobenzene (**1a**) (0.5 mmol), 83 mg of potassium carbonate (0.6 mmol), 73 mg of phenylboronic acid (**2a**) (0.6 mmol), and 5.5–22 mg of polyPy-Pd (0.63–2.52 mol % Pd) or 0.7 mg of palladium(II) acetate (0.63 mol % Pd). Then, a combination of 1.5 mL of the solvent [EGME, methanol, ethanol, dimethoxyethane (DME), acetonitrile, toluene, DCM, or propylene carbonate] and 0.5 mL of water was added and the mixture was stirred at room temperature on a magnetic stirrer. At specified time intervals (10 min, 1 h, and 4 h), approximately 100 μL of the reaction mixture was transferred into a vial containing water using a syringe. Then, 1 mL of ethyl acetate was added to the vial and the mixture was shaken. The organic layer was transferred to a small round-bottom flask and the solvent was evaporated under reduced pressure. The conversion was then determined from the ^1^H NMR spectrum of the crude residue based on iodobenzene (**1a**).^37^

### General Synthetic Procedure

A round-bottom flask was charged with 0.5 mmol iodobenzene derivative **1a–j**, 83 mg of potassium carbonate (0.6 mmol), phenylboronic acid derivative **2a–e** (0.6 mmol), and 22 mg of polyPy-Pd (2.52 mol % Pd). Then, 1.5 mL of EGME and 0.5 mL of water were added and the mixture was stirred for 24 h at room temperature. Ethyl acetate was then added to the crude reaction mixture and the heterogeneous catalyst was filtered off. The organic phase was washed three times with brine and 2 M KOH. The organic layer was then dried with sodium sulfate and the solvent was evaporated under reduced pressure. Where necessary, the product was further purified by column chromatography. The products were analyzed by ^1^H NMR. The ^1^H NMR spectra are in agreement with those from the literature.

#### 1,1′-Biphenyl (**3**) (71 mg, 92%)^37^

^1^H NMR (300 MHz, CDCl_3_) δ: 7.63–7.57 (m, 4H); 7.49–7.41 (m, 4H); 7.37–7.32 (m, 2H). ^13^C NMR (126 MHz, CDCl_3_) δ: 141.4, 128.9, 127.4, 127.3.

#### 4-Methoxy-1,1′-biphenyl (**4**) (82 mg, 88%)^37^

^1^H NMR (300 MHz, CDCl_3_) δ: 7.57–7.50 (m, 4H); 7.41 (t, *J* = 7.5 Hz, 2H); 7.30 (t, *J* = 7.3 Hz, 1H), 6.99 (d, *J* = 8.8 Hz, 2H); 3.86 (s, 3H). ^13^C NMR (126 MHz, CDCl_3_) δ: 159.3, 141.0, 133.9, 128.9, 128.3, 126.9, 126.8, 114.3, 55.5.

#### 3-Amino-1,1′-biphenyl (**5**) (49 mg, 57%)^38^

^1^H NMR (500 MHz, CDCl_3_) δ: 7.57 (dd, *J* = 8.2; 1.2 Hz, 2H); 7.42 (t, *J* = 7.6 Hz, 2H); 7.33 (tt, *J* = 7.4; 1.2 Hz, 1H); 7.23 (t, *J* = 7.8 Hz, 1H); 7.01 (d, *J* = 7.7 Hz, 1H); 6.96 (t, *J* = 2.0 Hz, 1H); 6.73 (ddd, *J* = 7.9; 2.3; 0.8 Hz, 1H). ^13^C NMR (126 MHz, CDCl_3_) δ: 142.7, 129.9, 128.8, 127.4, 127.4, 127.3, 118.5, 114.9, 114.7, 114.5.

#### 3-Methyl-1,1′-biphenyl (**6**) (78 mg, 93%)^15^

^1^H NMR (300 MHz, CDCl_3_) δ: 7.59 (d, *J* = 7.0 Hz, 2H); 7.487.30 (m, 6H); 7.17 (d, *J* = 7.2 Hz, 1H); 2.43 (s, 3H). ^13^C NMR (126 MHz, CDCl_3_) δ: 141.5, 141.4, 138.5, 128.9, 128.8, 128.1, 127.4, 127.3, 127.3, 124.4, 21.7.

#### 4-Chloro-1,1′-biphenyl (**8**) (82 mg, 87%)^37^

^1^H NMR (300 MHz, CDCl_3_) δ: 7.57–7.49 (m, 4H); 7.47–7.34 (m, 5H). ^13^C NMR (126 MHz, CDCl_3_) δ: 140.1, 139.8, 133.5, 129.0, 129.0, 128.5, 127.7, 127.1.

#### 3-Chloro-1,1′-biphenyl (**9**) (78 mg, 83%)^39^

^1^H NMR (300 MHz, CDCl_3_) δ: 7.57–7.55 (m, 3H); 7.48–7.32 (m, 6H). ^13^C NMR (126 MHz, CDCl_3_) δ: 143.2, 139.9, 134.8, 130.1, 129.0, 128.9, 128.0, 127.4, 127.4, 127.3, 127.3, 125.4.

#### 3-Nitro-1,1′-biphenyl (**10**) (81 mg, 81%)^37^

^1^H NMR (300 MHz, CDCl_3_) δ: 8.46 (t, *J* = 2.0 Hz, 1H); 8.21 (ddd, *J* = 8.2; 2.3; 1.0 Hz, 1H); 7.92 (ddd, *J* = 7.7; 1.7; 1.1 Hz, 1H); 7.65–7.59 (m, 3H); 7.53–7.41 (m, 3H). ^13^C NMR (126 MHz, CDCl_3_) δ: 147.8, 147.2, 138.9, 129.3, 129.1, 128.5, 127.9, 127.5, 124.5, 124.3.

#### 2,3,4,5,6-Pentafluoro-1,1′-biphenyl (**11**) (81 mg, 66%)^40^

^19^F NMR (471 MHz, CDCl_3_) δ: −143.77 (dd, *J* = 22.9; 8.2 Hz, 2F); −156.12 (t, *J* = 21.0 Hz, 1F); −162.76 (dt, *J* = 22.8; 8.2, 2F). ^1^H NMR (500 MHz, CDCl_3_) δ: 7.60 (d, *J* = 7.1 Hz, 1H); 7.52–7.34 (m, 5H). ^13^C NMR (126 MHz, CDCl_3_) δ: 141.4, 130.3, 129.4, 128.9, 128.9, 127.4, 127.3, 126.5.

#### 1,1′-Biphenyl-4-carboxylic Acid (**12**) (55 mg, 55%)^37^

^1^H NMR (300 MHz, DMSO) δ: 7.98 (d, *J* = 8.3 Hz, 2H); 7.67 (d, *J* = 7.1 Hz, 2H); 7.59 (d, *J* = 8.3 Hz, 2H); 7.46 (t, *J* = 7.4 Hz, 2H); 7.36 (t, *J* = 7.3 Hz, 1H). ^13^C NMR (126 MHz, DMSO) δ: 171.4, 141.2, 134.0, 129.9, 128.9, 127.5, 126.7, 125.7.

#### 4-*tert*-Butyl-1,1′-biphenyl (**7**) (95 mg, 87%)^41^

^1^H NMR (500 MHz, CDCl_3_) δ: 7.60 (dd, *J* = 8.3; 1.2 Hz, 2H), 7.55 (d, *J* = 8.6 Hz, 2H), 7.48 (d, *J* = 8.6 Hz, 2H), 7.44 (t, *J* = 7.7 Hz, 2H), 7.33 (t, *J* = 7.4 Hz, 1H); 1.37 (2s, 9H). ^13^C NMR (126 MHz, CDCl_3_) δ: 150.4, 141.2, 138.5, 128.8, 127.2, 127.1, 126.9, 126.8, 125.9, 125.8, 34.7, 31.5.

#### 4-((4-Methoxybenzyl)oxy)-1,1′-biphenyl (**13**) (112 mg, 75%)

^1^H NMR (500 MHz, CDCl_3_) δ: 7.56 (d, *J* = 7.1 Hz, 2H, C2′H, C6′H); 7.53 (d, *J* = 8.8 Hz, 2H, C2H, C6H); 7.42 (t, *J* = 7.7 Hz, 2H, C3′H, C5′H); 7.39 (d, *J* = 8.7 Hz, 2H, C2″H, C6″H); 7.31 (t, *J* = 7.4 Hz, 1H, C4′H); 7.05 (d, *J* = 8.8 Hz, 2H, C3H, C5H); 6.94 (d, *J* = 8.7 Hz, 2H, C3″H, C5″H); 5.04 (s, 2H, CH_2_); 3.83 (s, 3H, CH_3_). ^13^C NMR (126 MHz, CDCl_3_) δ: 159.6 (C4″), 158.6 (C4), 141.0 (C1′), 134.1 (C1), 129.4 (C3″, C4″), 129.1 (C1″), 128.9 (C3′, C5′), 128.3 (C3, C5), 126.9 (C2′, C6′), 126.8 (C4′), 115.3 (C2, C6), 114.2 (C2″, C6″), 70.0 (CH_2_), 55.5 (CH_3_). ATR FTIR (neat) ν (cm^–1^): 1606, 1513, 1485, 1465, 1378, 1242, 1196, 1176, 1113, 1032, 1021, 1004, 870, 823, 758.

HRMS (ES-): calcd for C_20_H_18_O_2_, *m*/*z* = 289.1234; found, *m*/*z* = 289.1245.

#### 1,1′-Biphenyl-4-carbonitrile (**14**) (79 mg, 88%)^42^

^1^H NMR (300 MHz, CDCl_3_) δ: 7.71 (q, *J* = 8.7 Hz, 4H); 7.59 (dd, *J* = 8.2; 1.4 Hz, 2H); 7.51–7,42 (m, 3H). ^13^C NMR (126 MHz, CDCl_3_) δ: 145.8, 139.3, 132.7, 129.2, 128.8, 127.9, 127.4, 119.1, 111.0.

#### 4-Nitro-1,1′-biphenyl (**15**) (66 mg, 66%)^43^

^1^H NMR (300 MHz, CDCl_3_) δ: 8.31 (d, *J* = 8.9 Hz, 2H); 7.74 (d, *J* = 8.9 Hz, 2H); 7.63 (dd, *J* = 8.1; 1.5 Hz, 2H); 7.53–7.42 (m, 3H). ^13^C NMR (126 MHz, CDCl_3_) δ: 147.8, 147.2, 138.9, 129.3, 129.1, 127.9, 127.5, 124.2.

## Conclusions

A polyHIPE pyridine polymer could be used as a solid support for Pd catalysis. The reactivity of the solid-supported catalyst remains high and enables the synthesis of various biphenyls from iodobenzene and phenylboronic acid derivatives. The use of EGME as a solvent is necessary to prevent the inactivation of Pd through the formation of palladium black. For efficient reuse of the polyPy-Pd, the solvents for isolation must be carefully selected to prevent leaching of the Pd from the solid support. Direct conversion of phenylboronic acid to biphenyl is also possible in a single step by combining the iodination of phenylboronic acid **2a** to iodobenzene **1a** with the in situ Suzuki–Miyaura coupling of product **1a** with the starting compound **2a**.

## Supporting Information Available

Additional experimental procedures and NMR spectra of compounds (PDF)

## Abbreviations

HIPE: high internal phase emulsion
EGME: ethylene glycol monomethyl ether
DCM: dichloromethane
DME: dimethoxyethane
DEC: diethyl carbonate
