# Supplementary material for: Reusable Pd-PolyHIPE for Suzuki–Miyaura Coupling
Source: ACS Omega. 2022 Apr 6;7(15):12610–6. doi: 10.1021/acsomega.1c06318 (PMC9026024; doi:10.1021/acsomega.1c06318)
Supplement: Supplementary file 1 — ao1c06318_si_001.pdf [file ao1c06318_si_001.pdf]

Supporting Information

Reusable Pd-PolyHIPE for Suzuki-Miyaura Coupling

Miha Ravbar[1], Amadeja Koler[2], Muzafera Paljevac[2], Peter Krajnc[2], Mitja Kolar[1], Jernej Iskra[1*]

[1]University of Ljubljana, Faculty of Chemistry and Chemical Technology, Večna pot 113, 1000 Ljubljana, Slovenia.
[2]University of Maribor, Faculty of Chemistry and Chemical Engineering, Smetanova ulica 17, 2000 Maribor, Slovenia.

Table of Contents

1. General information 3

2. Procedures .... **Napaka! Zaznamek ni definiran.**

3. NMR Spectra 8

   3.1. 1,1'-biphenyl 8

   3.2. 4-methoxy-1,1'-biphenyl 9

   3.3. 3-amino-1,1'-biphenyl 10

   3.4. 3-methyl-1,1'-biphenyl 11

   3.5. 4-*tert*-butyl-1,1'-biphenyl 12

   3.6. 4-chloro-1,1'-biphenyl 13

   3.7. 3-chloro-1,1'-biphenyl 14

   3.8. 3-nitro-1,1'-biphenyl 15

   3.9. 2,3,4,5,6-pentafluoro-1,1'-biphenyl 16

   3.10. 1,1'-biphenyl-4-carboxylic acid 17

   3.11. 4-((4-methoxybenzyl)oxy) -1,1'-biphenyl 18

   3.12. 1,1'-biphenyl-4-carbonitrile 21

   3.13. 4-nitro-1,1'-biphenyl 22

4. References 23

1. General information

Iodobenzene (Fluka), phenylboronic acid (FluoroChem), potassium carbonate (Fluka), palladium(II) acetate (FluoroChem), 4-chloroiodobenzene (Fluka), 4-iodoanisole (Sigma-Aldrich), 3-iodoaniline (Sigma-Aldrich), 3-iodotoluene, 4-iodobenzoic acid (Koch-Light), 1-iodo-3-nitrobenzene (Sigma-Aldrich), 4-*tert*-butyliodobenzene (Sigma-Aldrich), 3-chloroiodobenzene (Sigma-Aldrich), 2,3,4,5,6-pentafluoroiodobenzene (FluoroChem), 4-cyanophenylboronic acid (Sigma-Aldrich), 4-(4'-methoxybenzyloxy)phenylboronic acid (Sigma-Aldrich), 4-*tert*-butylphenylboronic acid (FluoroChem), 4-nitrophenylboronic acid (FluoroChem), iodine (Kemika), 37% hydrochloric acid (Honeywell), 40% hydrogen peroxide, sodium sulphite (Merck), Pd standard 1000 µg/mL (J.T. Baker)
Poly(pyridine-co-divinylbenzene) was prepared according to the procedure in the literature.[1] Elemental analysis was used to determine the amount of 4-vinylpyridine to be 1,1 mmol/g polymer.

EGME (2-methoxyethanol, FluoroChem), methanol (LabExpert), ethanol (Honeywell), DME (dimethoxyethane, Fluka), acetonitrile (Honeywell), toluene (LabExpert), dichloromethane (Honeywell), propylene carbonate (Millipore), chloroform (Scharlan), chloroform-d (Eurisotop), DMSO-d6 (Eurisotop), 2-methyltetrahydrofurane (Sigma-Aldrich), trifluorotoluene (Fluka), ethyl acetate (Honeywell)

NMR spectra were taken using BRUKER DPX 300 NMR spectrometer and BRUKER AVANCE III 500 MHz NMR spectrometer. Chemical shifts are given in reference to TMS (^{1}H: $\delta = 0$ ppm), $CHCl_3$ (^{1}H: $\delta = 7.26$ ppm, ^{13}C: $\delta = 77.2$ ppm) or DMSO (^{1}H: $\delta = 2.5$ ppm, ^{13}C: $\delta = 39.5$ ppm) for ^{1}H or ^{13}C spectra and $CFCl_3$ (^{19}F: $\delta = 0$ ppm) for ^{19}F spectra.

The amount of palladium on the polymer was determined using Varian AA240 atomic absorption spectrometer ($\lambda = 247.6$ nm, $\Delta\lambda = 0{,}2$ nm, $\Phi_{(air)} = 3.5$ L/min, $\Phi_{(C_2H_2)} = 1.5$ L/min, $i_{(hollow\ cathode)} = 5$ mA).

2. Determining the amount of palladium on the polymer:

A small amount of catalyst was weighed into a long-necked Kjeldahl flask, typically in the range of 5 to 25 mg. Then 1 mL of conc. HNO_3 and 2 mL of conc. H_2SO_4 were added. The mixture was heated over a burner flame until no more gas escaped. The mixture was then cooled, 1 mL of 30% H_2O_2 was added, and the mixture was heated again until it became clear. If necessary, 1 mL 30% H_2O_2 was added, and the mixture was briefly heated again. The clear liquid was then transferred to a 25 mL volumetric flask and the flask was filled to the mark with 2% HNO_3. A blank sample was prepared using the same procedure, but without catalyst. Palladium standard solutions were typically prepared at 1 mg/L, 2 mg/L, 3 mg/L, 4 mg/L by serial dilution of the commercial palladium standard Pd standard (Carlo Erba 0.999 ug/ml, $\rho = 1.022$ g/ml). The absorbance was measured for all prepared solutions using the AAS Varian AA 240 instrument with the settings: $\lambda = 247.6$ nm, $\Delta\lambda = 0.2$ nm, i(HC) = 5 mA, $\Phi(C_2H_2) = 1.5$ mL/min, Φ(AIR) = 3.5 mL/min. The palladium concentration in the samples was determined

using a calibration curve. The palladium content in the weighed samples was then determined from this palladium concentration.

3. A reuse of the catalyst

Procedure A – washing the polymer with MeOH:

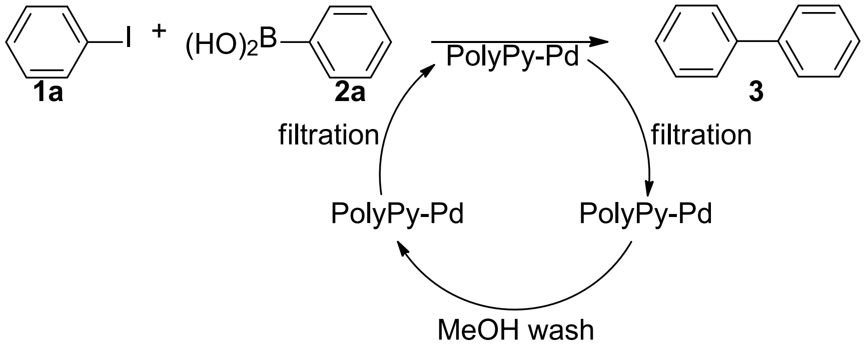

A round bottom flask was charged with 408 mg of iodobenzene (**1a**) (2 mmol), 332 mg of potassium carbonate (2.4 mmol), 292 mg of phenylboronic acid (**2a**) (2.4 mmol) and 88 mg of polyPy-Pd (2.52 mol% Pd). Then, 6 mL of EGME and 2 mL of water were added and the mixture was stirred on a magnetic stirrer at room temperature. Reaction was followed by taking aliquots at specified times as described in the General procedure for optimization reactions. After 24 h, polyPy-Pd was filtered off from the reaction mixture and then stirred for 1 h in methanol. It was then filtered off from the methanol, dried under air and used in a subsequent reaction following the same procedure as described above. The whole procedure was repeated for the third time. The results are shown in Table 4.

Procedure B – catalyst reuse with intermediate regeneration:

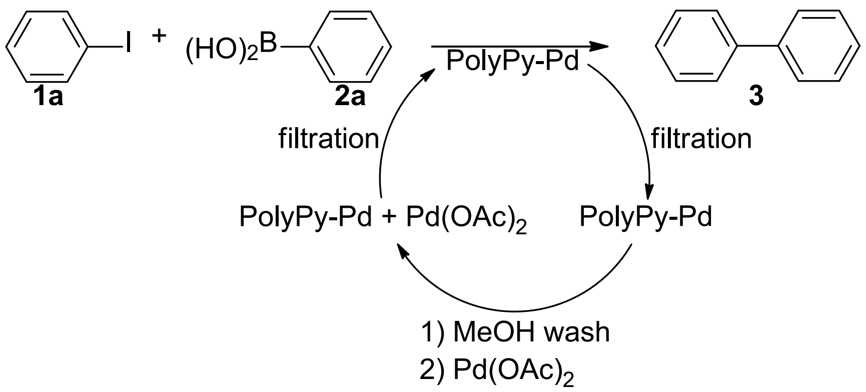

A round bottom flask was charged with 408 mg of iodobenzene (**1a**) (2 mmol), 332 mg of potassium carbonate (2.4 mmol), 292 mg of phenylboronic acid (**2a**) (2.4 mmol) and 88 mg of polyPy-Pd (2.52 mol% Pd). Then, 6 mL of EGME and 2 mL of water were added and the mixture was stirred on a magnetic stirrer at room temperature. The progress of the reaction was followed by taking aliquots at specific times, as described in the General procedure for

optimization reactions. After 24 h, polyPy-Pd was filtered off from the reaction mixture and then stirred in methanol for 1 h, followed by filtration and air drying. Before reuse, the catalyst was stirred in a solution of palladium acetate in acetonitrile (35 mg $Pd(OAc)_2$ in 5 mL of MeCN) and then filtered and dried again. This catalyst was then reused in a subsequent reaction using the same procedure as described above. The entire procedure was then repeated for the third time. The results are shown in Table 4.

Procedure C – substitution of EGME with DCM or $PhCH_3$:

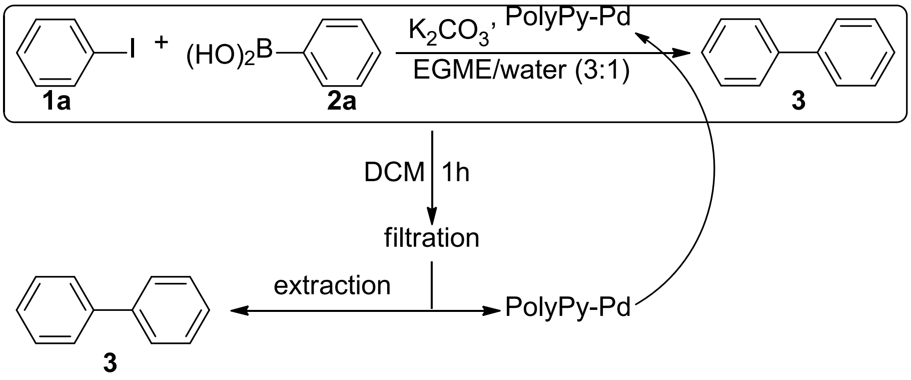

The reaction was carried out as in Procedure A, except that toluene or DCM was used instead of EGME.

Procedure D – catalyst reuse with the addition of DCM after reaction:

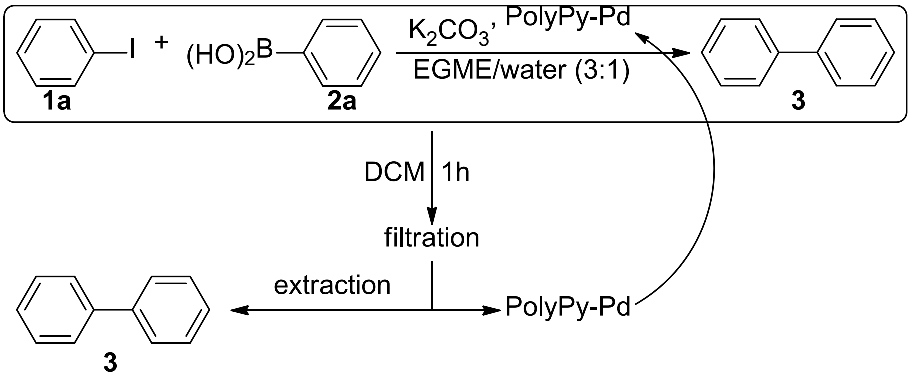

A round bottom flask was charged with 204 mg of iodobenzene (**1a**) (1 mmol), 166 mg of potassium carbonate (1.2 mmol), 146 mg of phenylboronic acid (**2a**) (1.2 mmol) and 44 mg of polyPy-Pd (2.52 mol% Pd). Then, 3 mL of EGME and 1 mL of water were added and the mixture was stirred on a magnetic stirrer at room temperature. After 4 h, 10 mL of dichloromethane was added to the reaction mixture and stirred for another 1 h. PolyPy-Pd was filtered from the reaction mixture and washed with dichloromethane. PolyPy-Pd was dried under air and then used in a subsequent reaction by charging a round bottom flask with 102 mg of iodobenzene (**1a**) (0.5 mmol), 83 mg of potassium carbonate (0.6 mmol), 73 mg of phenylboronic acid (**2a**) (0.6 mmol) and 22 mg of polyPy-Pd from the previous reaction. Then, 1.5 mL of EGME and 0.5 mL of water were added to the flask and the mixture was stirred at

room temperature on a magnetic stirrer. The reaction course and final conversion were determined as in Procedure A. The results are shown in Table 4.

Procedure E – catalyst reuse with addition of DEC after reaction:

Prior to, polyPy-Pd was stirred overnight in EGME, then filtered and air dried. A round bottom flask was charged with 204 mg of iodobenzene (**1a**) (1 mmol), 166 mg of potassium carbonate (1.2 mmol), 146 mg of phenylboronic acid (**2a**) (1.2 mmol) and 88 mg of polyPy-Pd (2.52 mol% Pd). Then, 3 mL of EGME and 1 mL of water were added and the mixture was stirred on a magnetic stirrer at room temperature. The progress of the reaction was followed by taking aliquots at specific times, as described in the General procedure for optimization reactions. After 24 h, 10 mL of diethyl carbonate (DEC) was added to the reaction mixture and stirred for additional 1 h. PolyPy-Pd was filtered from the reaction mixture and washed with diethyl carbonate. PolyPy-Pd was dried under air and then used in a subsequent reaction, using the same procedure as described above. The results are displayed in Table 4.

Determination of activity of polyPy-Pd after washing it with the reaction solvent:
In a round bottom flask, 22 mg of polyPy-Pd, 1.5 mL of EMGE and 0.5 mL of water were stirred for 24 h at room temperature. PolyPy-Pd was then filtered off and both, the polymer and mother liquor were used for Suzuki-Miyaura coupling reaction as described below in procedure 1 and 2, respectively.

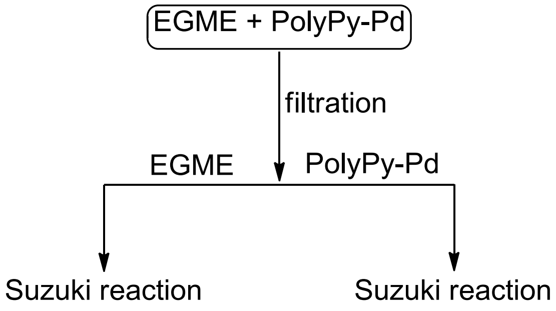

1) Reaction with mother liquor: A round bottom flask was carged with 102 mg of iodobenzene (**1a**) (0.5 mmol), 83 mg of potassium carbonate (0.6 mmol) and 73 mg of phenylboronic acid (**2a**) (0.6 mmol). The mother liquor from above was then added to the flask and the mixture was stirred at room temperature on a magnetic stirrer. At specified intervals (10 min, 1 h and 4 h), approximately 100 µL of the reaction mixture was transferred to a vial containing water using a syringe. Then, 1 mL of ethyl acetate was added to the vial and the mixture was shaken. The organic layer was transferred to a small round bottom flask and the solvent was evaporated under reduced pressure. The conversion was determined from the ^1H NMR spectrum of the crude residue based on iodobenzene (**1a**).

2) Reaction with washed polymer: A round bottom flask was charged with 60 mg of iodobenzene (**1a**) (0.29 mmol), 49 mg of potassium carbonate (0.35 mmol), 43 mg of phenylboronic acid (**2a**) (0.35 mmol) and the 13 mg of washed polyPy-Pd from above. Then, 1 mL of EGME and 0.33 mL of water were added and the mixture was stirred at room temperature on a magnetic stirrer. At specified intervals (10 min, 1 h and 4 h), approximately 100 µL of the reaction mixture was transferred to a vial containing water using a syringe. Then, 1 mL of ethyl

acetate was added to the vial and the mixture was shaken. The organic layer was transferred to a small round bottom flask and the solvent was evaporated under reduced pressure. The conversion was determined from the ^{1}H NMR spectrum of the crude residue based on iodobenzene (**1a**).

Iodination of phenylboronic acid:

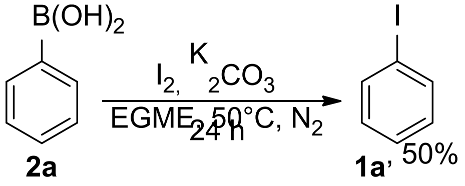

A reaction vial was filled with 61 mg of phenylboronic acid (**2a**) (0.5 mmol), 138 mg of potassium carbonate (1 mmol) and 191 mg of iodine (0.75 mmol). Then, 3 mL of EGME was added and the vial was equipped with a nitrogen balloon. The reaction mixture was stirred at 50 °C for 24 h on a magnetic stirrer. After the reaction, ethyl acetate was added to the reaction mixture and the organic layer was washed three times with brine, 2M KOH and a solution of sodium sulfite. The organic layer was dried with sodium sulphate and the solvent was evaporated under reduced pressure. The product was analysed by ^{1}H NMR. The ^{1}H NMR spectrum of the product is in agreement with the spectrum of commercial iodobenzene.

Product: iodobenzene (**1a**) (51 mg, 50%)
^{1}H NMR (300 MHz, $CDCl_3$) δ: 7.70 (dd, *J*=8.3; 1.2 Hz, 2H); 7.33 (tt, *J*=7.5; 1.1 Hz, 1H); 7.10 (t, *J*=7.7 Hz, 2H).

Iodination of phenylboronic acid and Suzuki reaction in one-step:

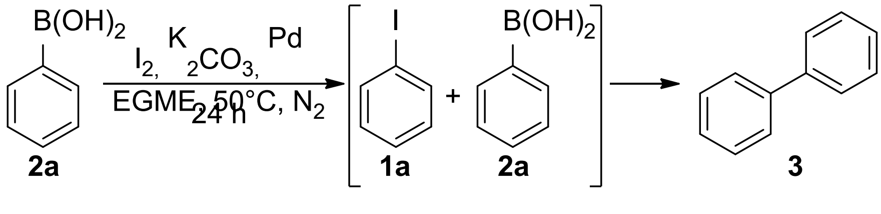

A reaction vial was filled with 122 mg of phenylboronic acid (**2a**) (1 mmol), 138 mg of potassium carbonate (1 mmol), 127 mg of iodine (0.5 mmol) and 5.6 mg of palladium(II) acetate (0.025 mmol) or 22 mg of polyPy-Pd (2.52 mol% Pd). Then, 3 mL of EGME was added and the vial was equipped with a nitrogen balloon. The reaction mixture was stirred at 50 °C for 24 h on a magnetic stirrer- After the reaction, ethyl acetate was added to the reaction mixture (in the case of polyPy-Pd, the catalyst was first removed from the reaction mixture by filtration) and the organic layer was washed three times with brine, 2M KOH and a solution of sodium sulfite. The organic layer was dried with sodium sulphate and the solvent was evaporated under reduced pressure. The product was analysed by ^{1}H NMR. The results are shown in Scheme 1. ^{1}H NMR spectra of the product is in agreement with the one from literature.[2]

4. NMR Spectra

4.1. 1,1'-biphenyl 3

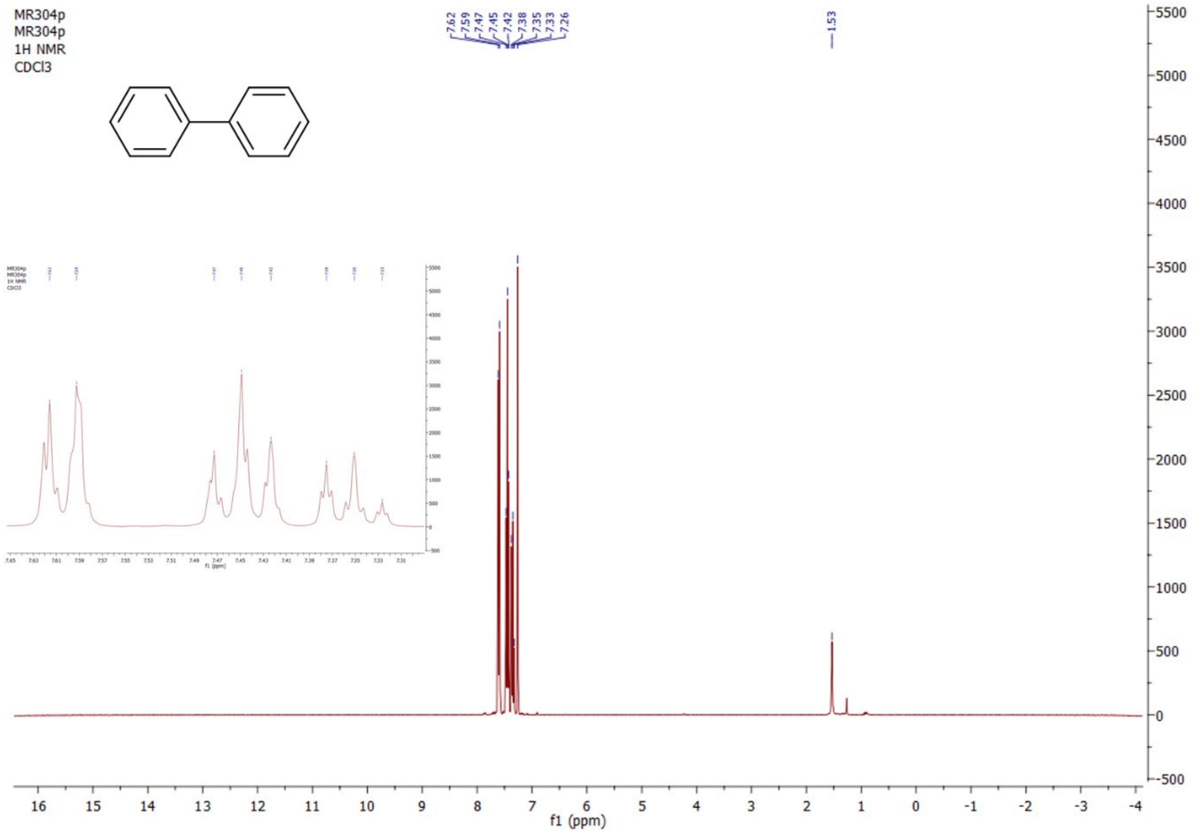

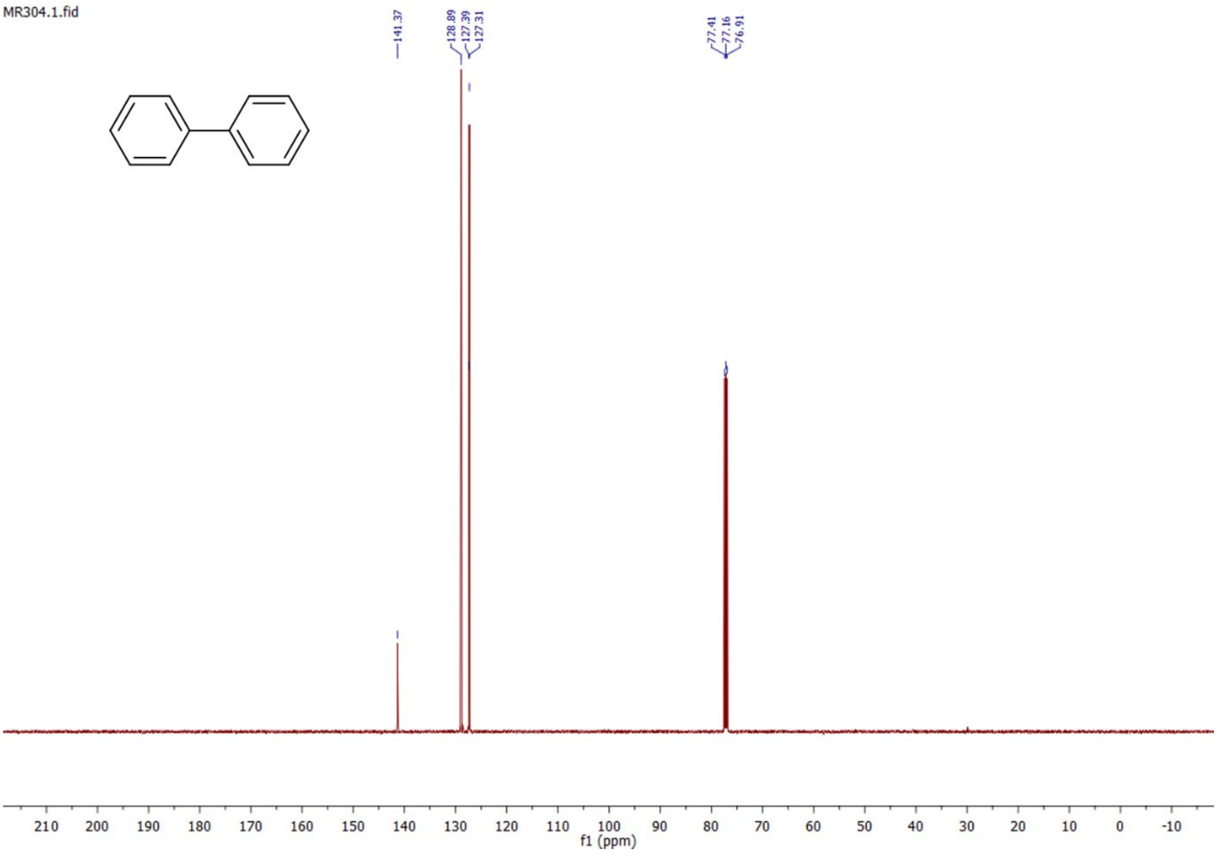

4.2. 4-methoxy-1,1'-biphenyl 4

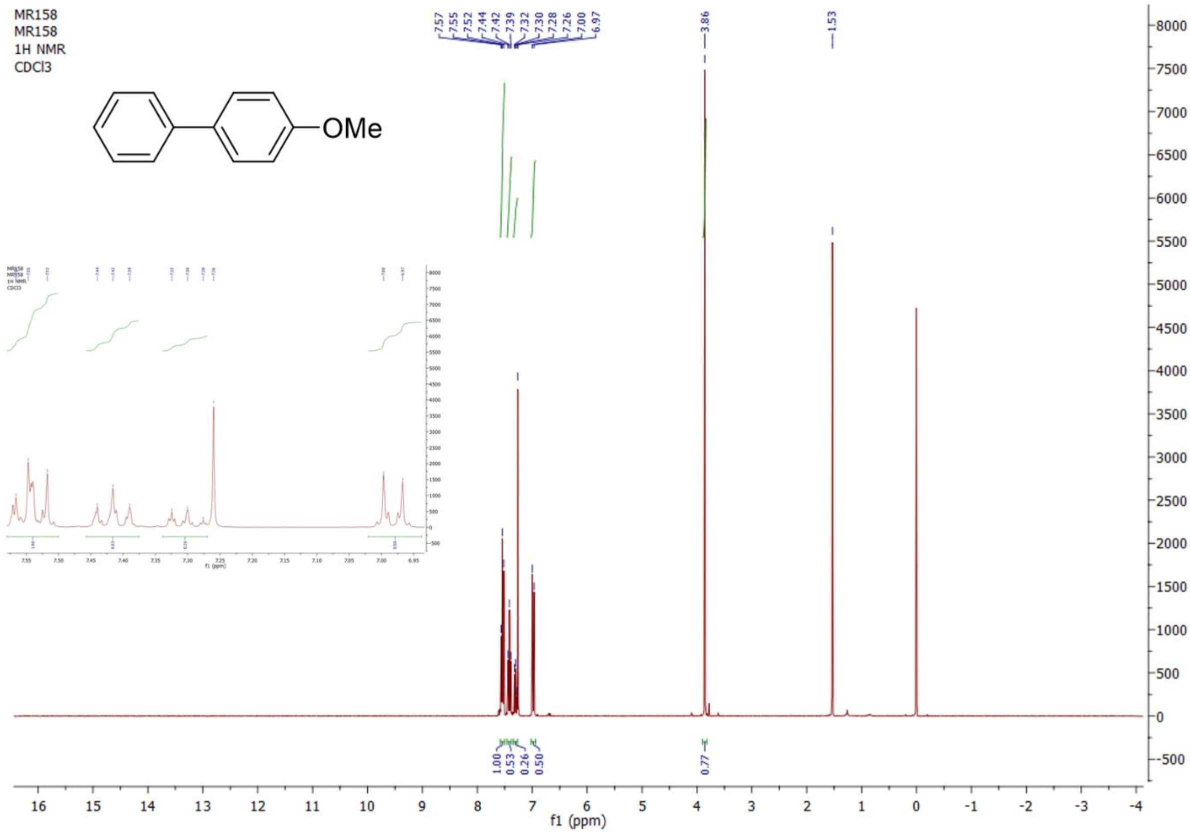

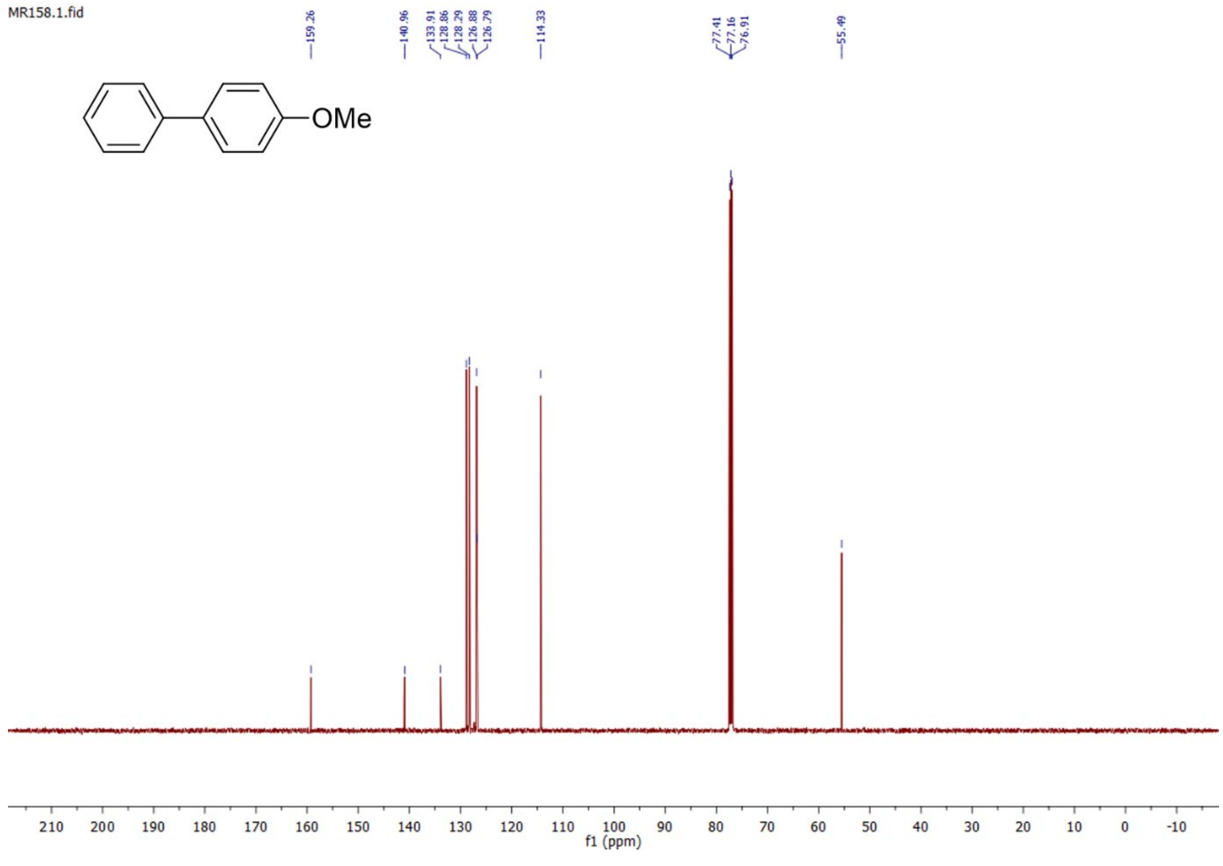

4.3. 3-amino-1,1'-biphenyl 5

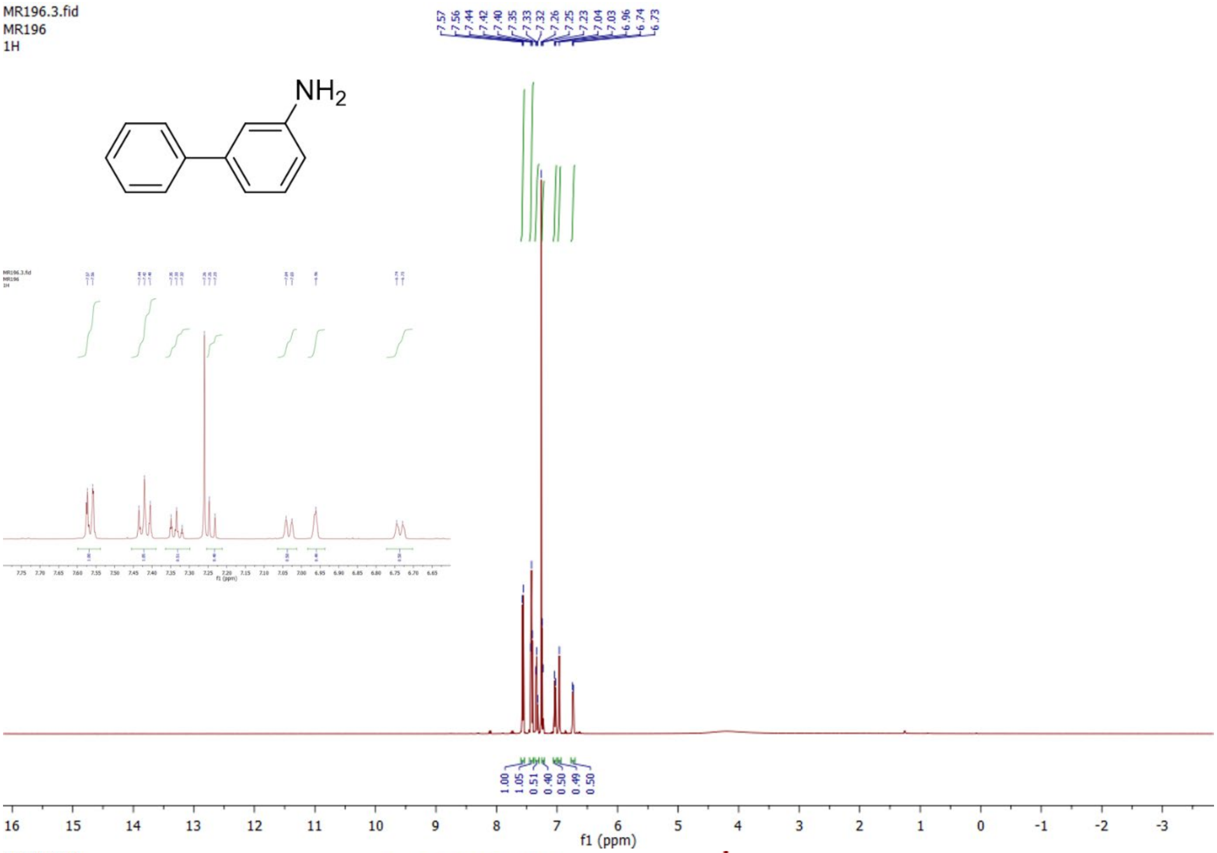

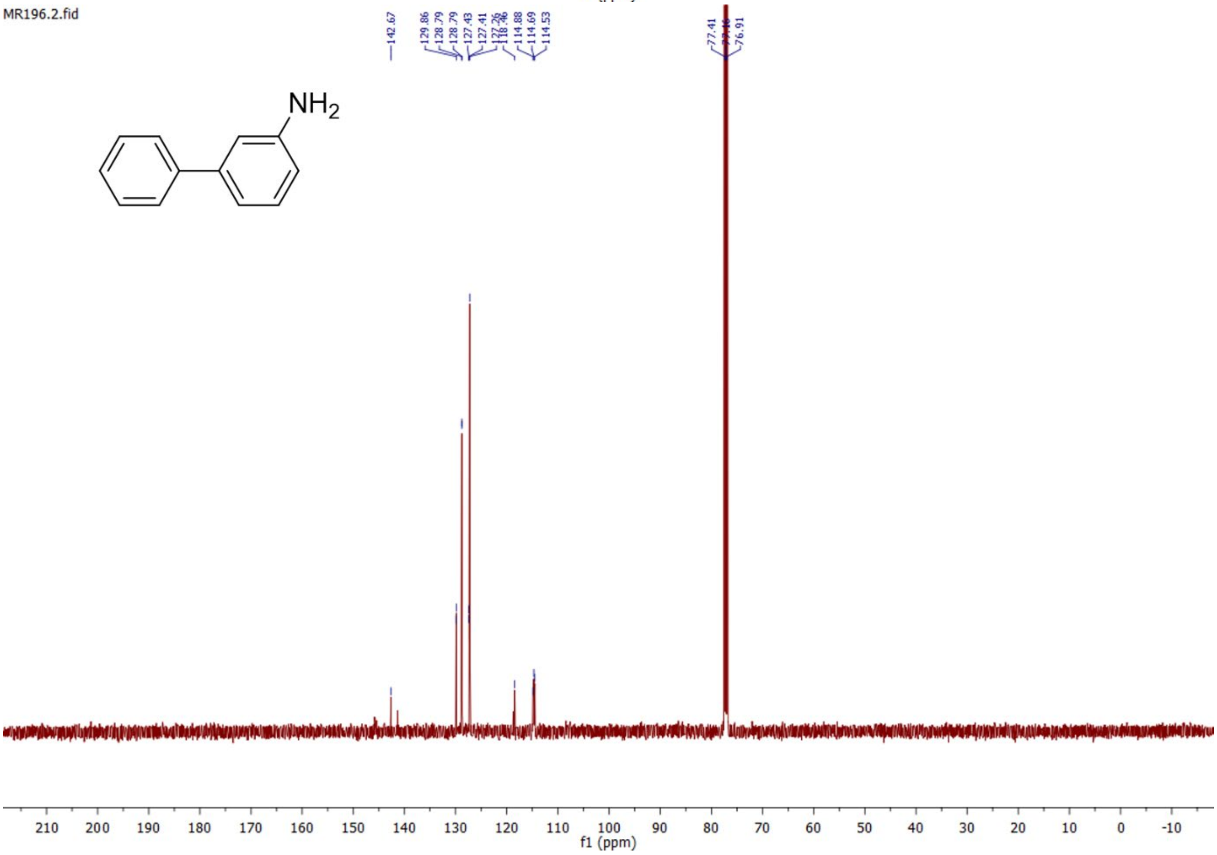

4.4. 3-methyl-1,1'-biphenyl 6

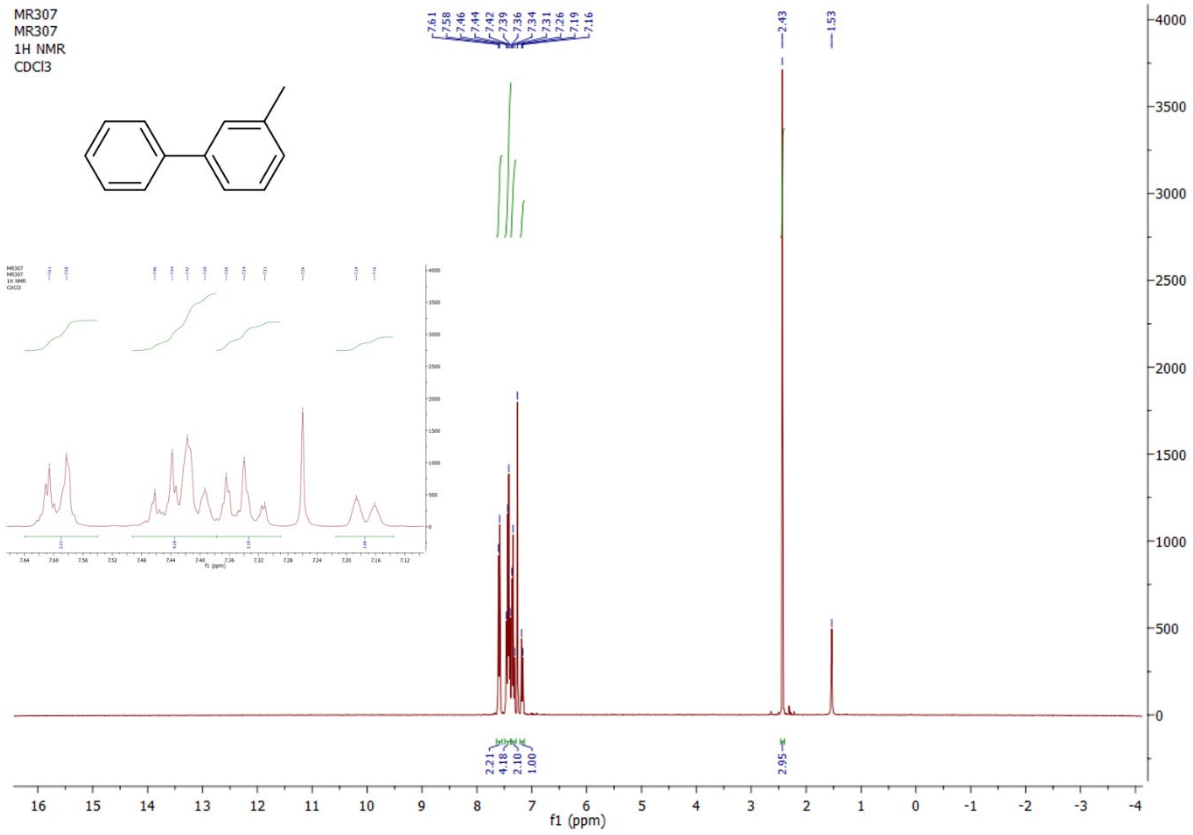

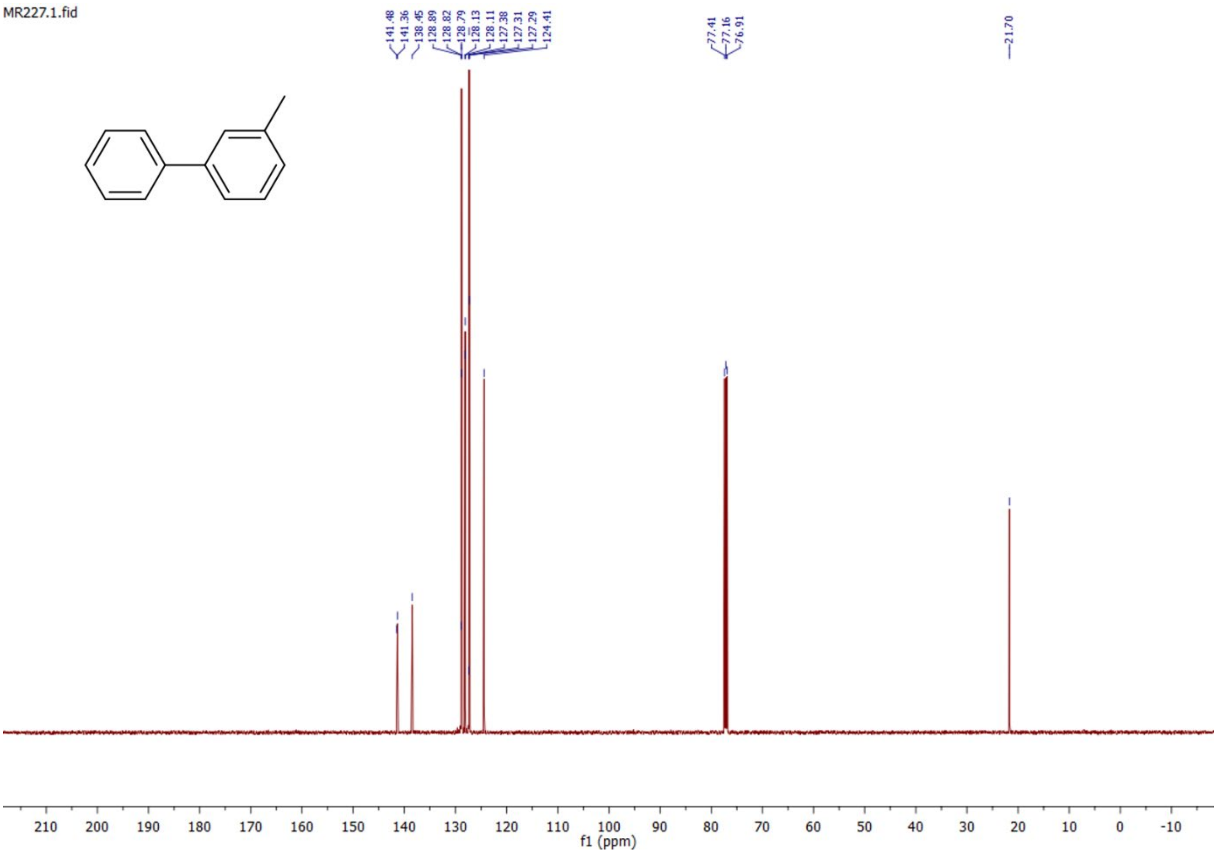

4.5. 4-*tert*-butyl-1,1'-biphenyl 7

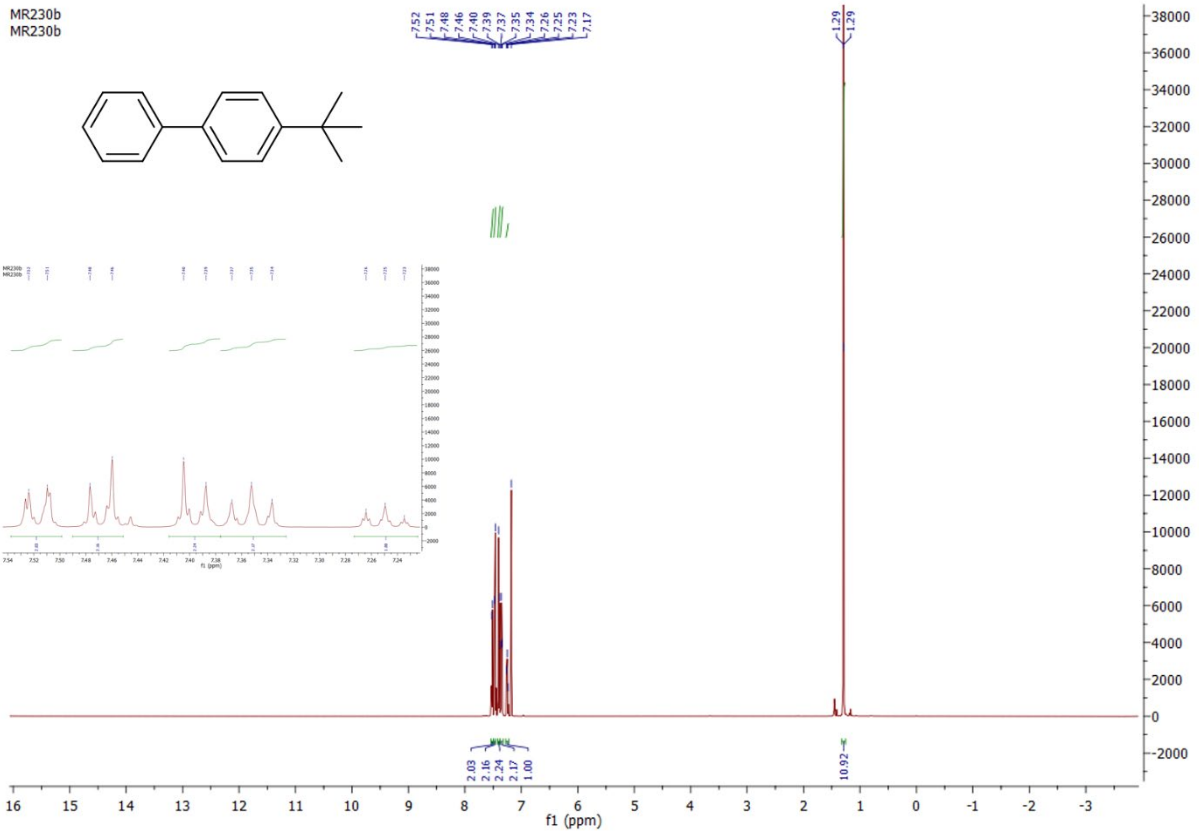

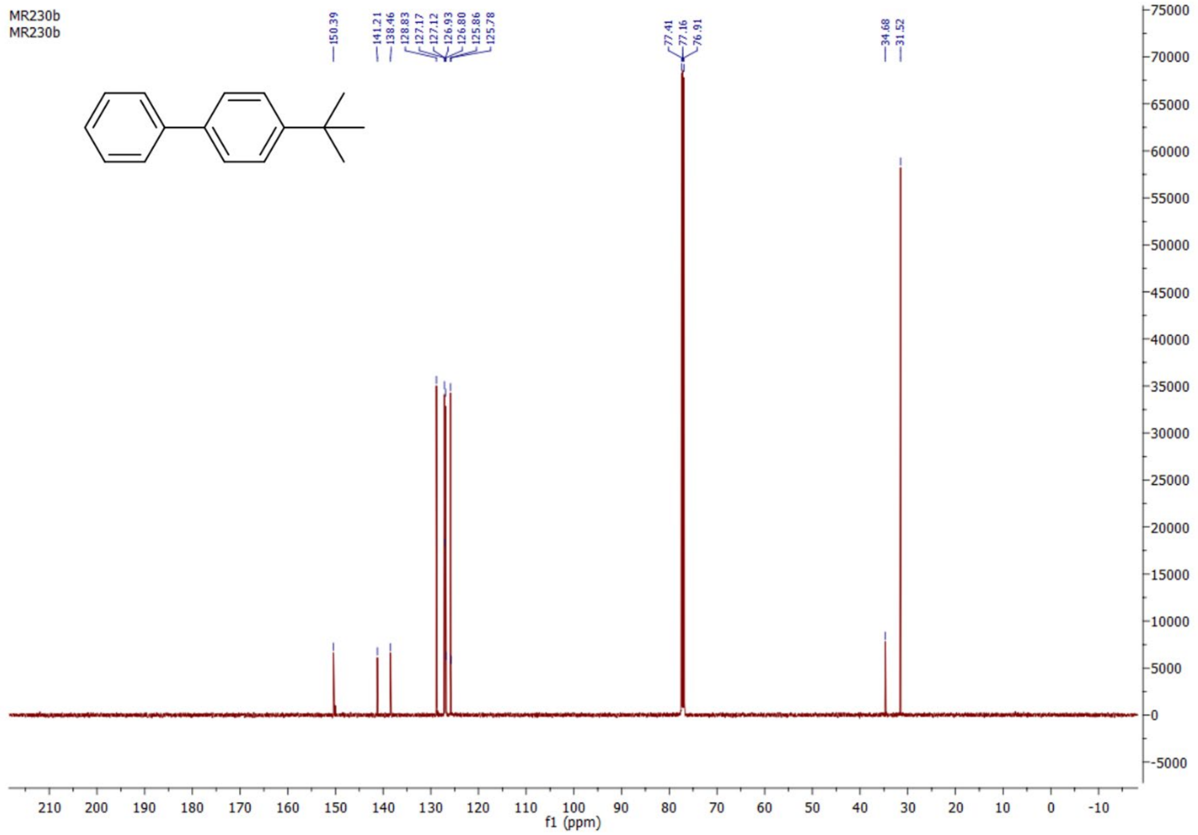

4.6. 4-chloro-1,1'-biphenyl 8

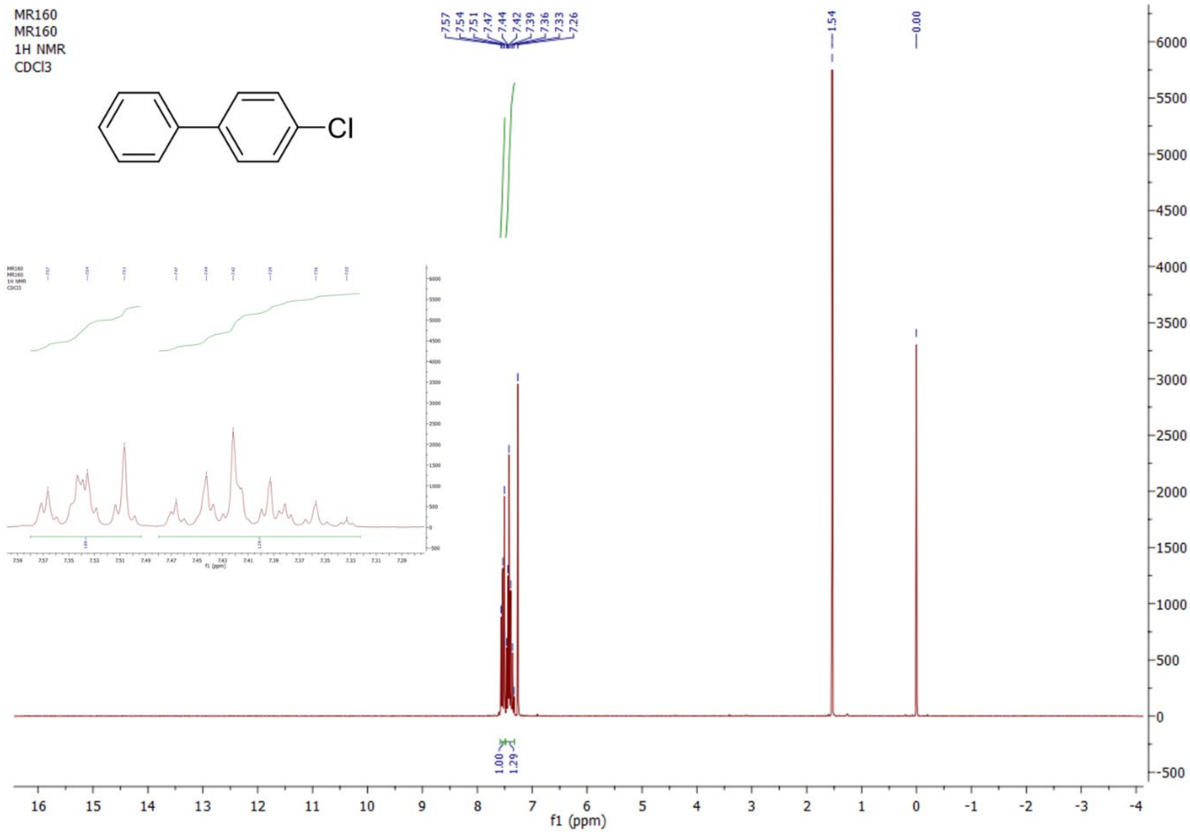

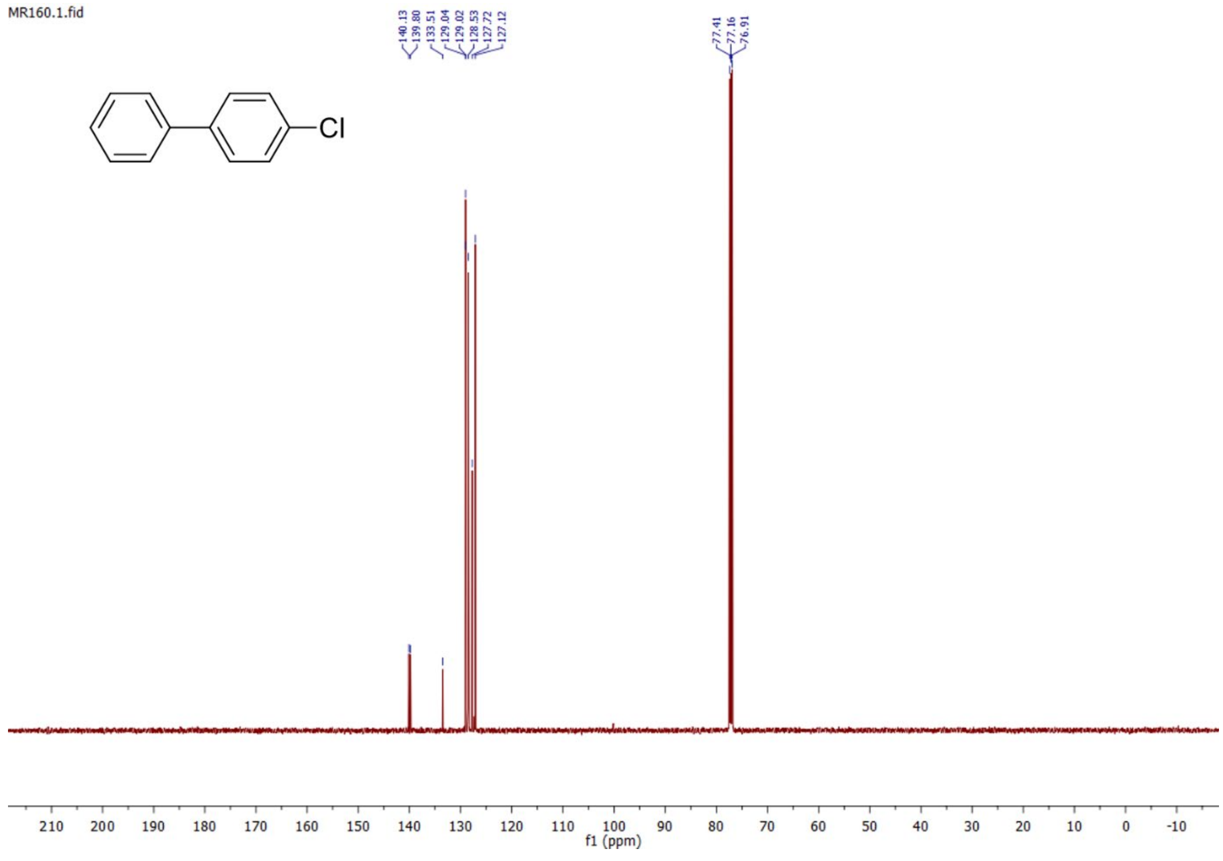

4.7. 3-chloro-1,1'-biphenyl 9

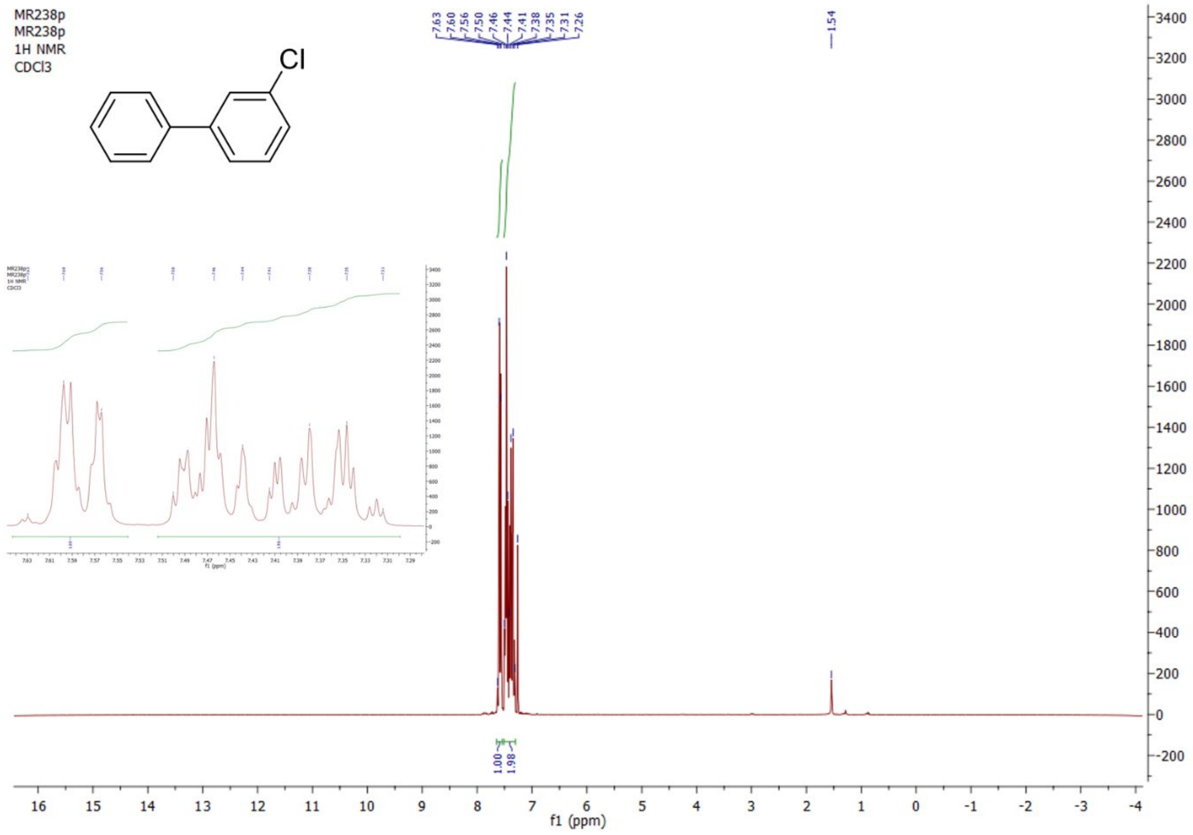

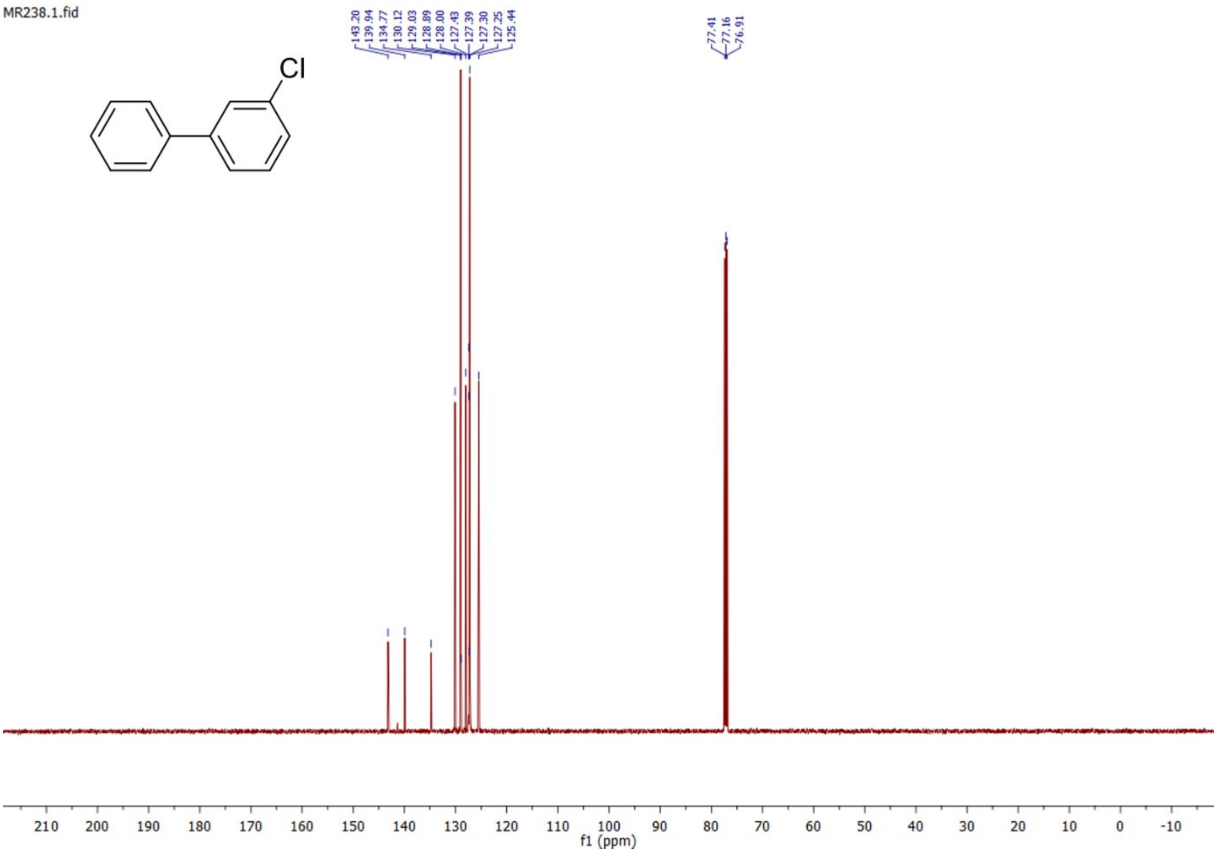

4.8. 3-nitro-1,1'-biphenyl 10

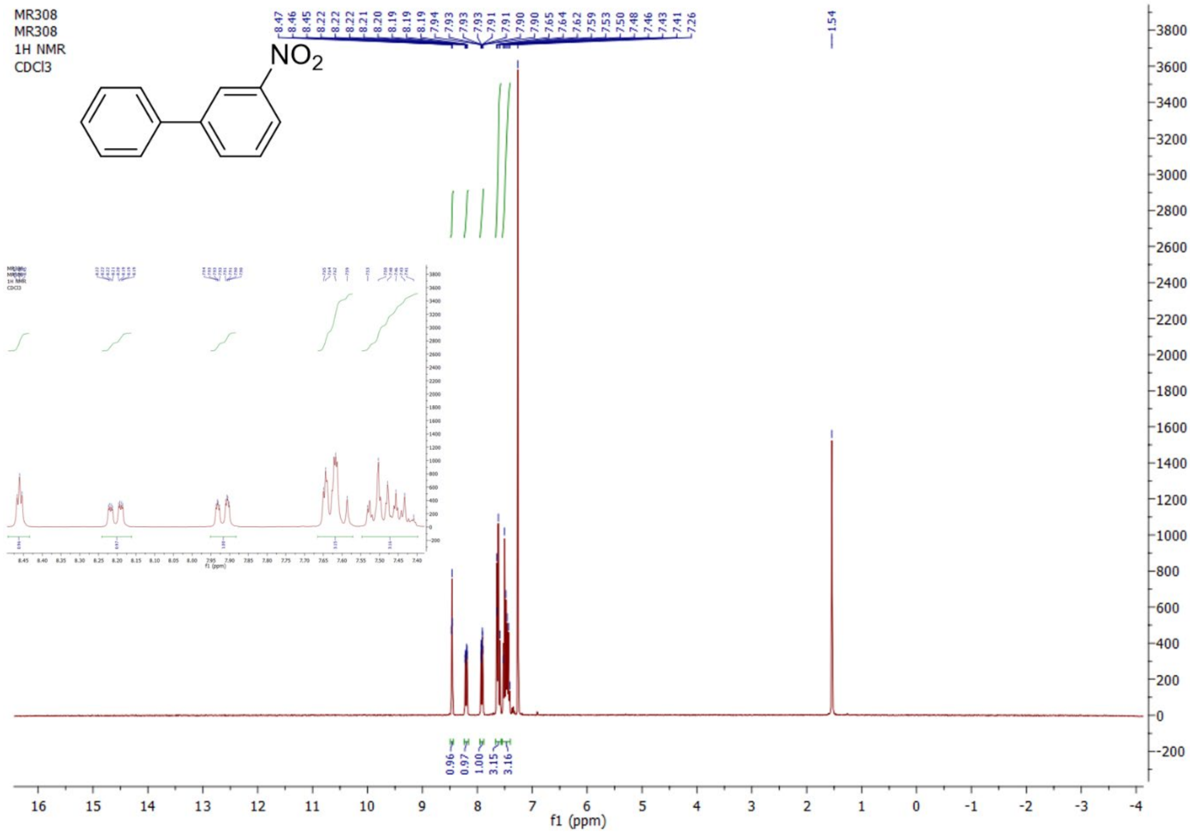

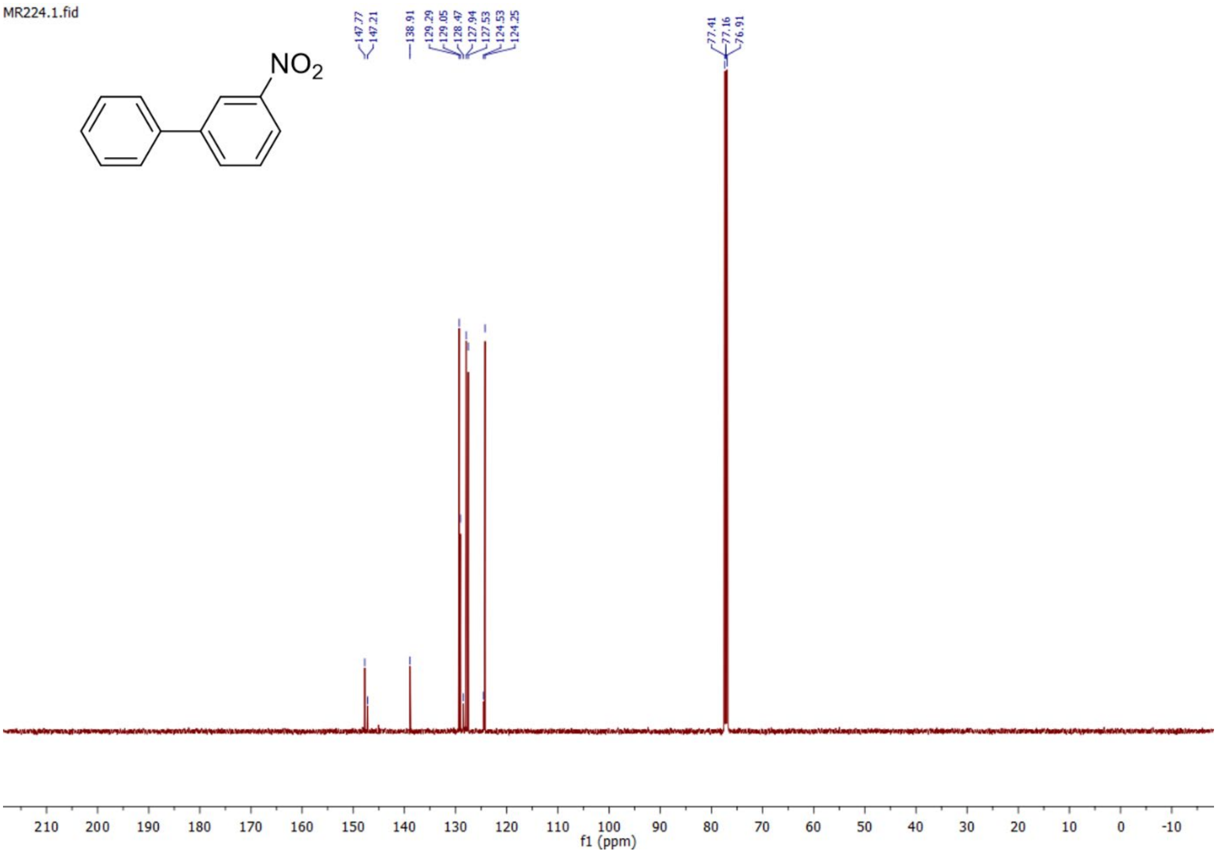

4.9. 2,3,4,5,6-pentafluoro-1,1'-biphenyl 11

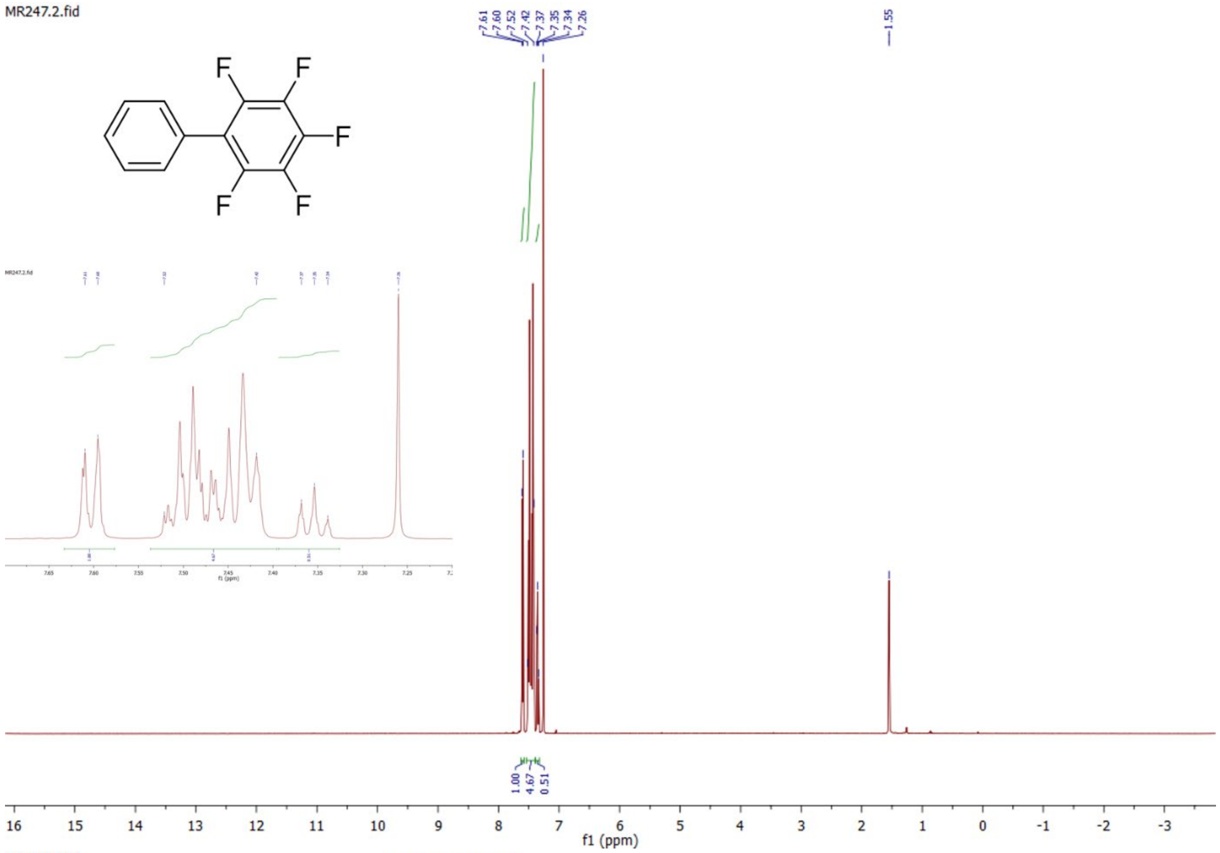

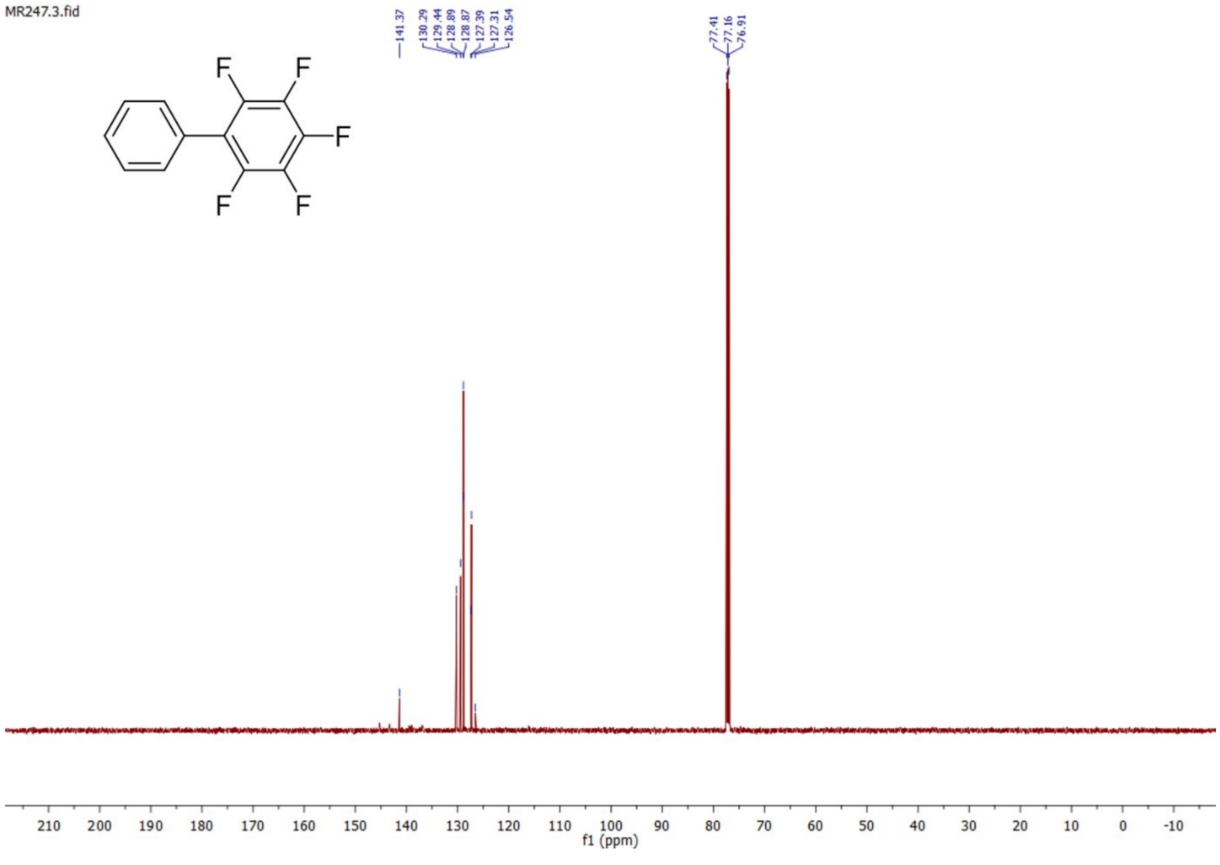

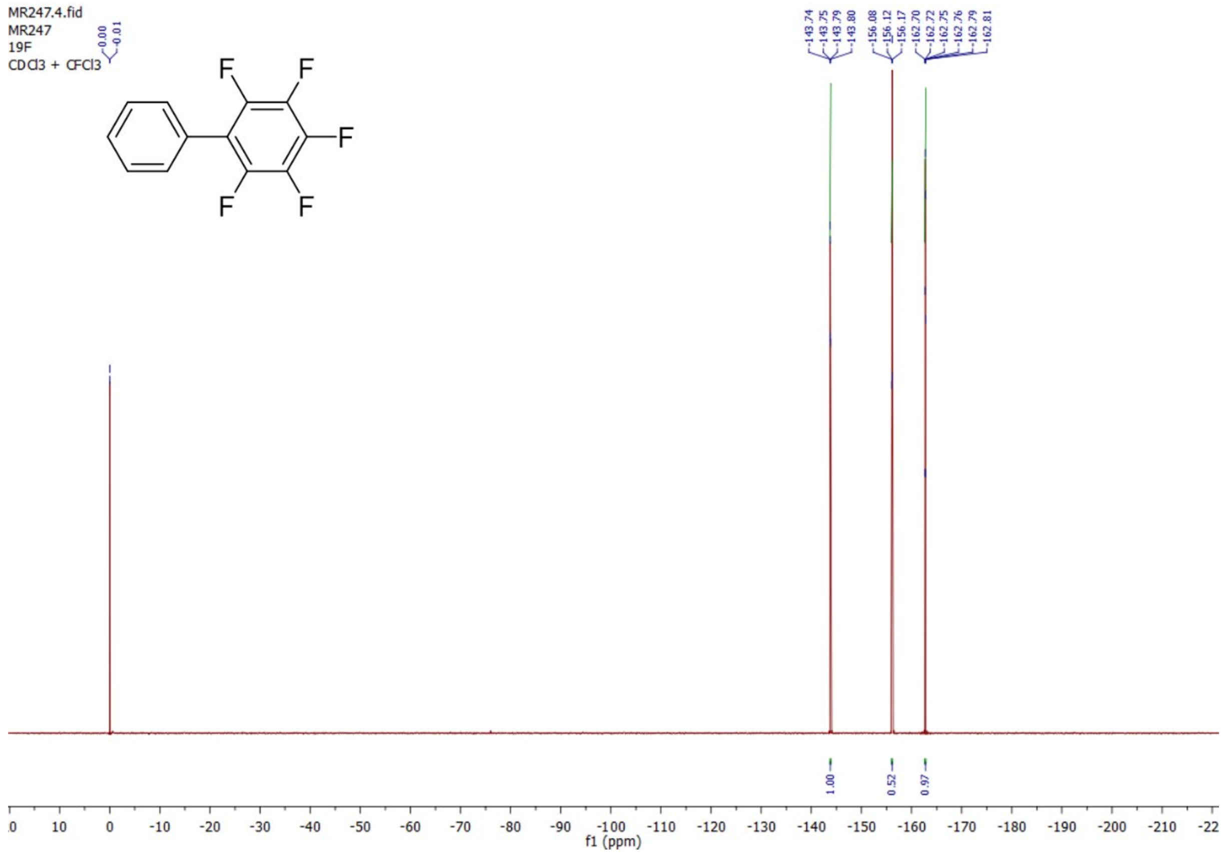

4.10. 1,1'-biphenyl-4-carboxylic acid 12

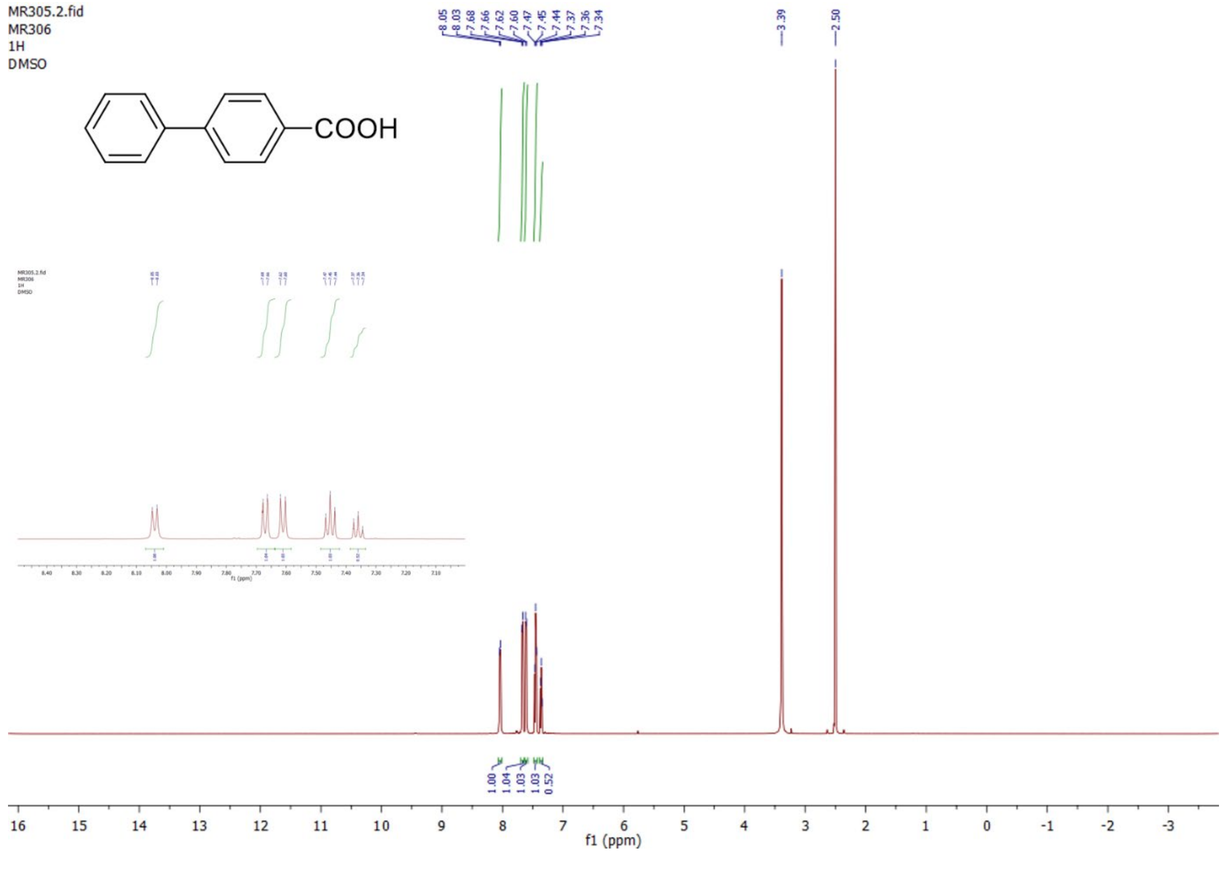

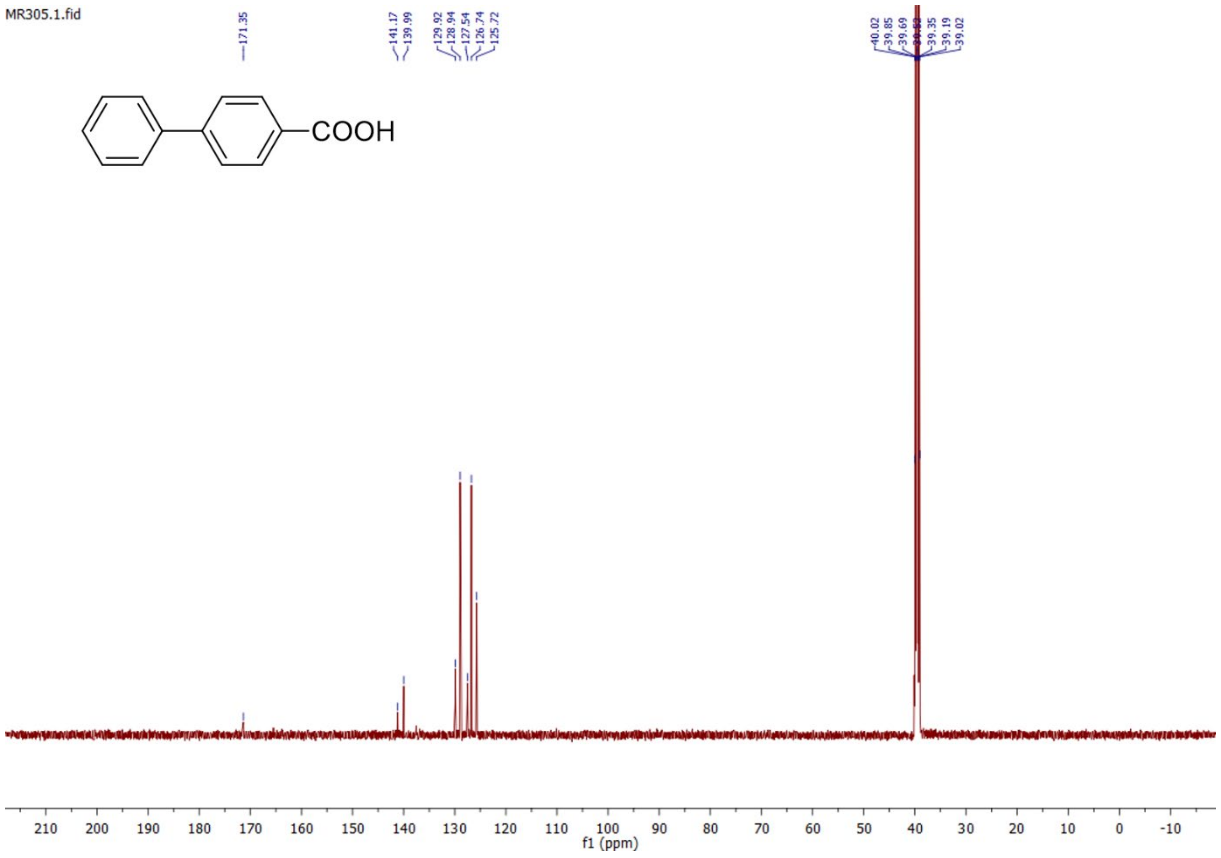

4.11. 4-((4-methoxybenzyl)oxy) -1,1'-biphenyl 13

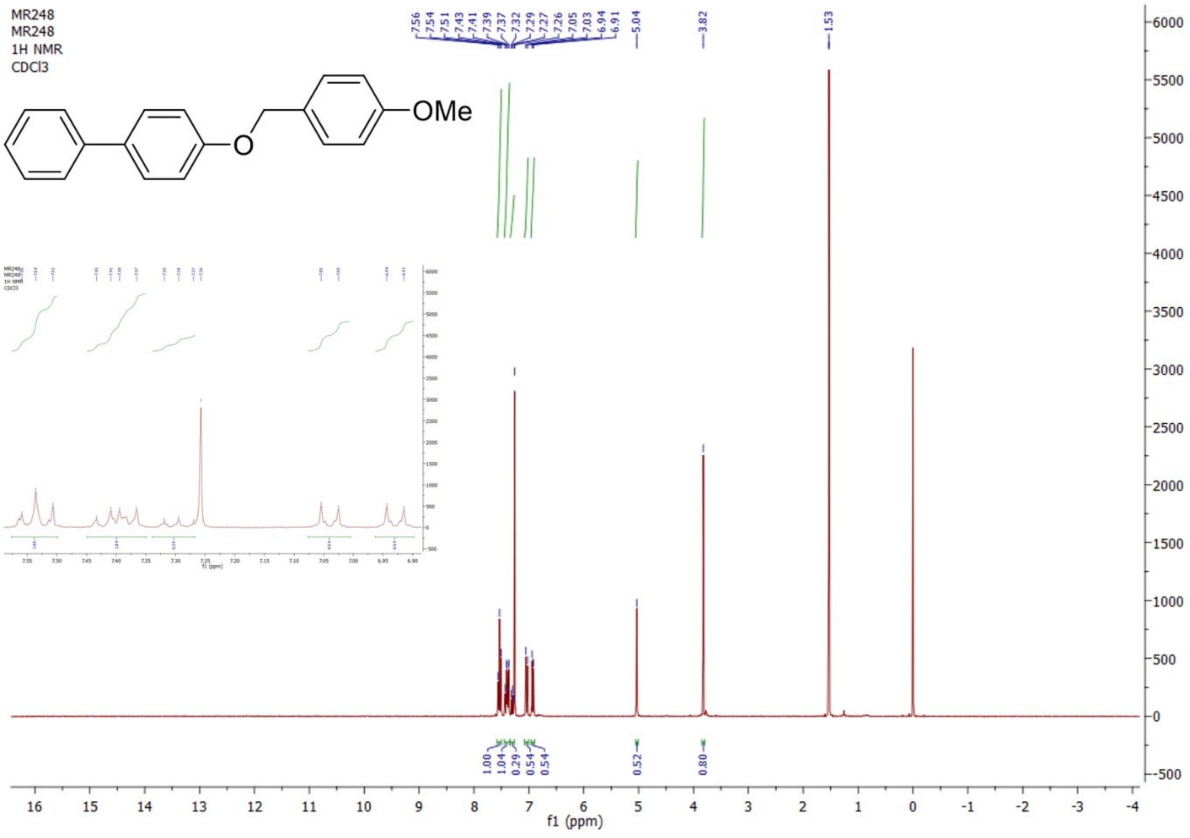

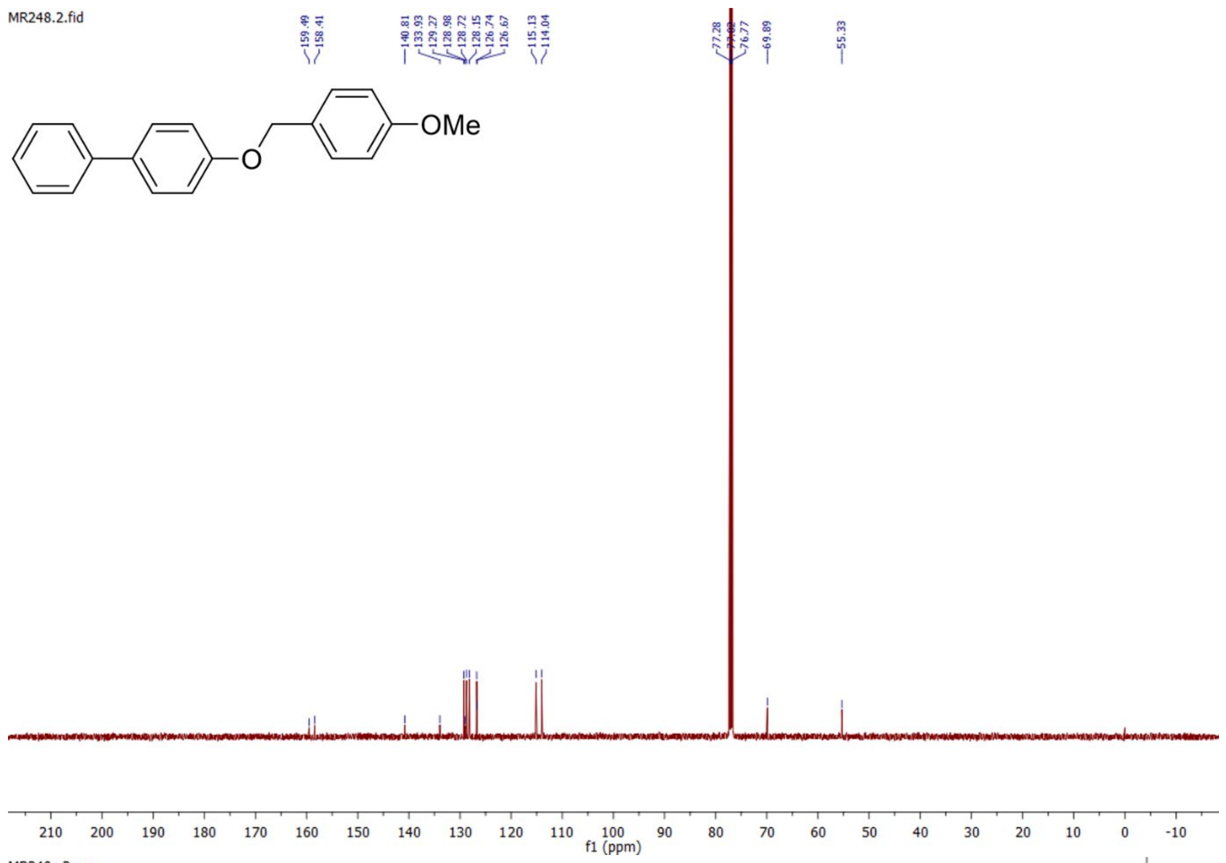
MR248.2.fid
159.49
158.41
140.81
133.93
129.27
128.98
128.72
128.15
126.74
126.67
115.13
114.04
77.28
76.77
69.89
55.33
O
OMe
f1 (ppm)

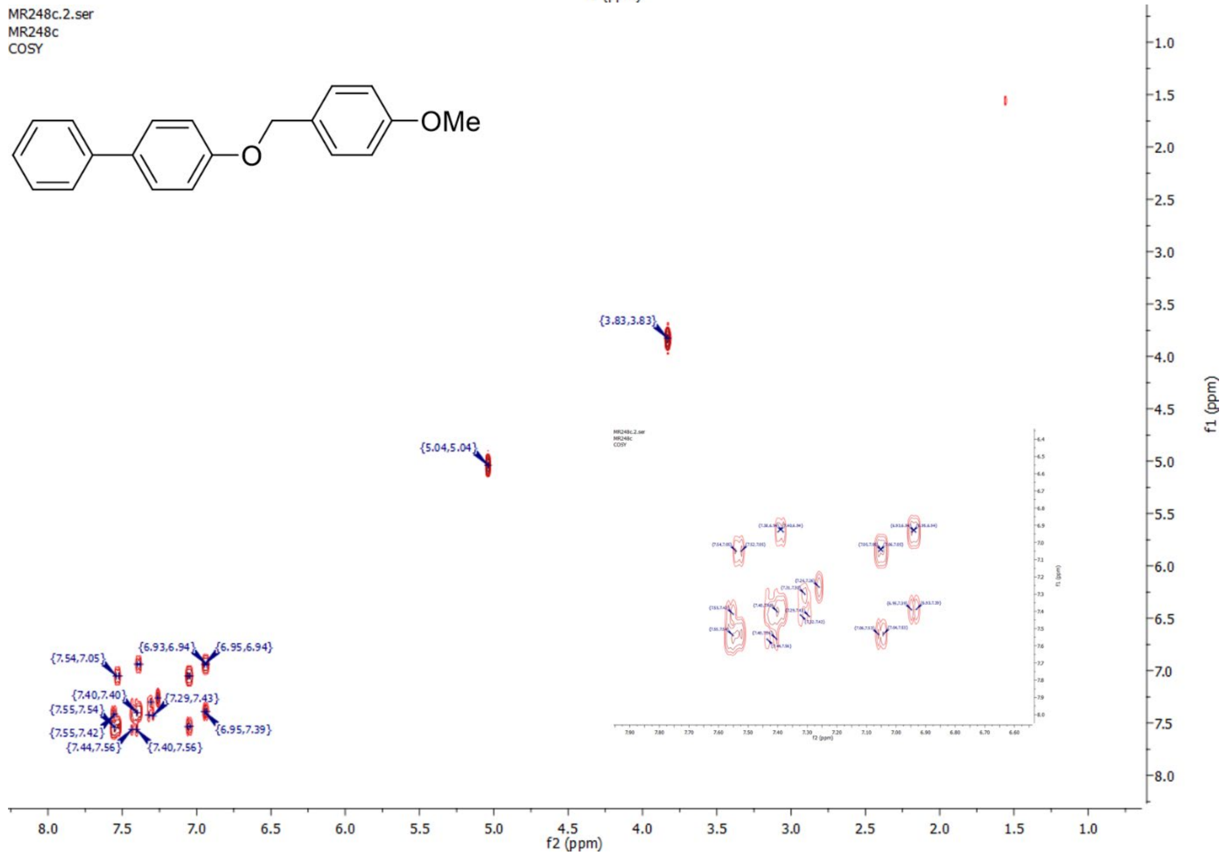
MR248c.2.ser
MR248c
COSY
O
OMe
{3.83,3.83}
{5.04,5.04}
{7.54,7.05}
{6.93,6.94}
{6.95,6.94}
{7.40,7.40}
{7.29,7.43}
{7.55,7.54}
{7.55,7.42}
{6.95,7.39}
{7.44,7.56}
{7.40,7.56}
f2 (ppm)
f1 (ppm)

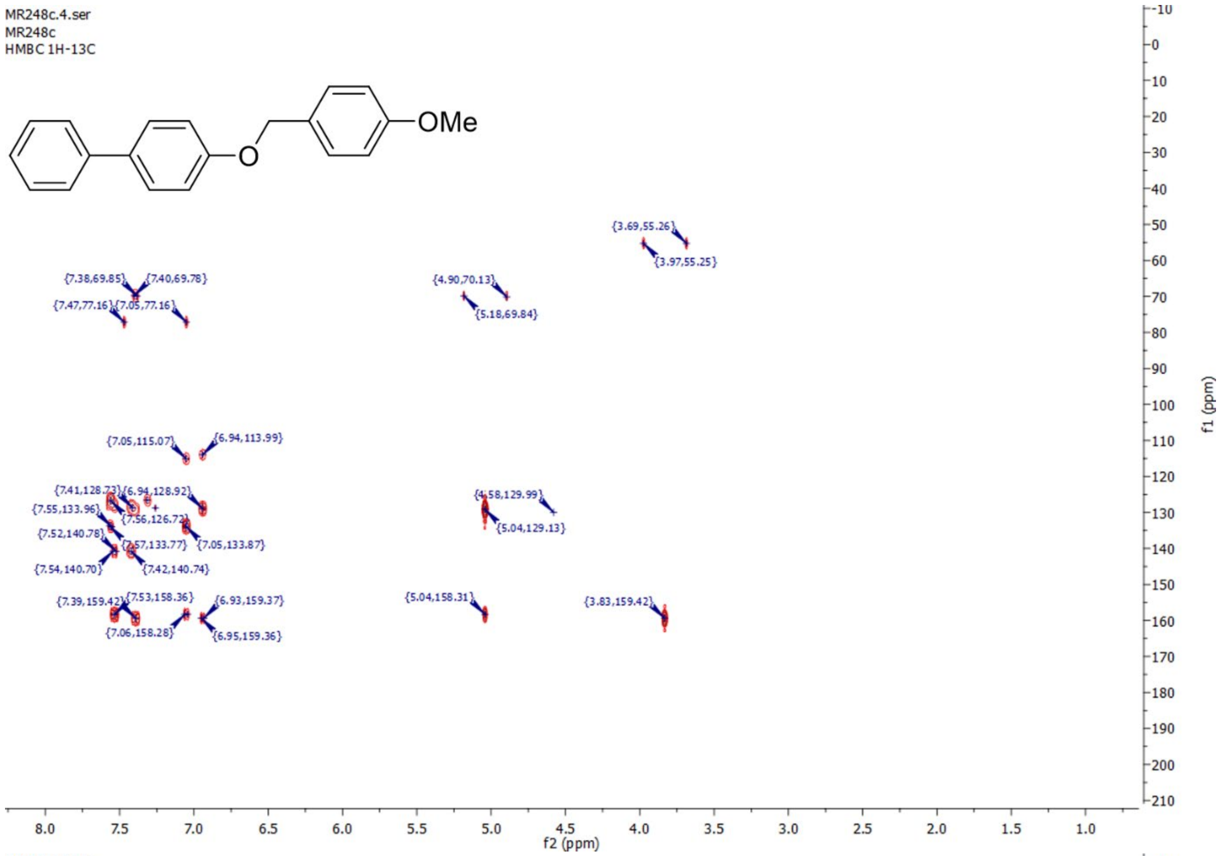
MR248c.4.ser
MR248c
HMBC 1H-13C
OMe
O
{3.69,55.26}
{3.97,55.25}
{7.38,69.85}
{7.40,69.78}
{7.47,77.16}
{7.05,77.16}
{4.90,70.13}
{5.18,69.84}
{7.05,115.07}
{6.94,113.99}
{7.41,128.73}
{6.94,128.92}
{7.55,133.96}
{7.56,126.72}
{7.52,140.78}
{7.57,133.77}
{7.05,133.87}
{7.54,140.70}
{7.42,140.74}
{4.58,129.99}
{5.04,129.13}
{7.39,159.42}
{7.53,158.36}
{6.93,159.37}
{7.06,158.28}
{6.95,159.36}
{5.04,158.31}
{3.83,159.42}
f1 (ppm)
f2 (ppm)

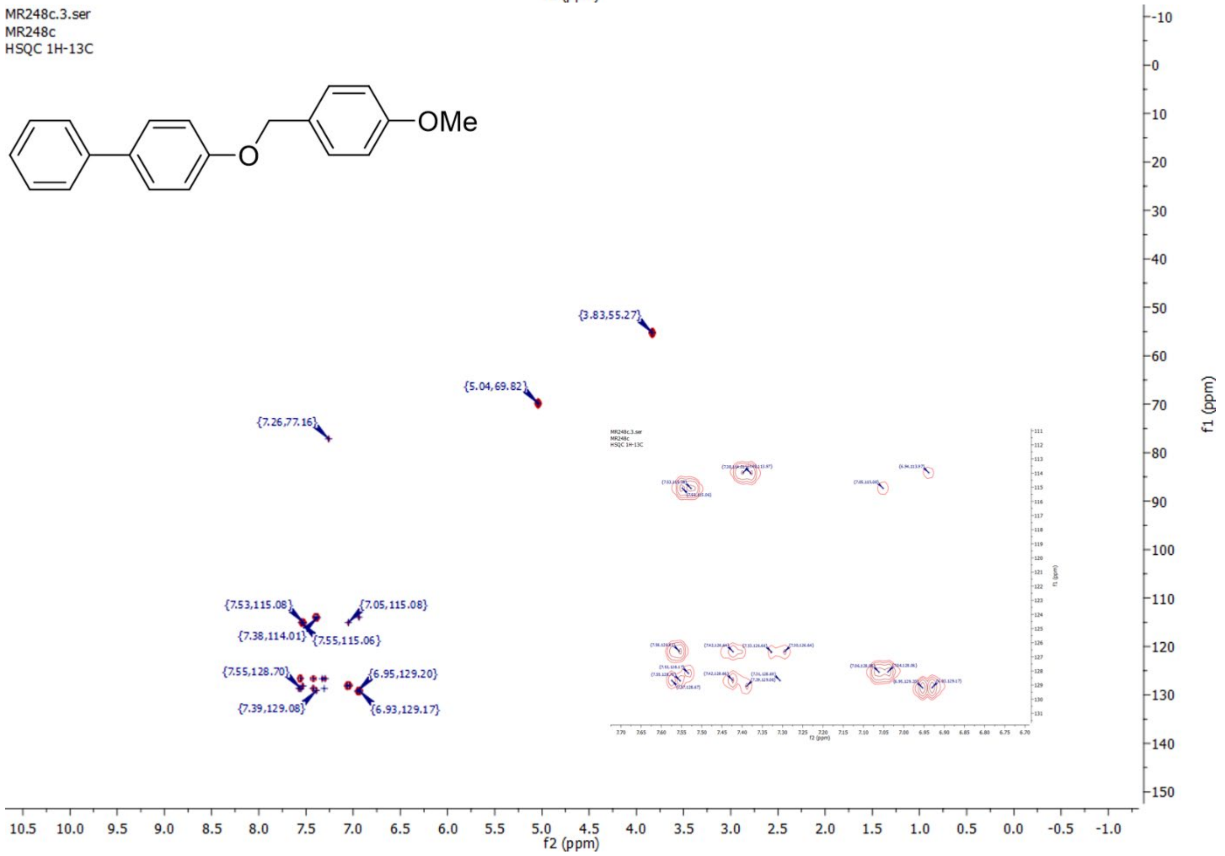
MR248c.3.ser
MR248c
HSQC 1H-13C
OMe
O
{3.83,55.27}
{5.04,69.82}
{7.26,77.16}
{7.53,115.08}
{7.05,115.08}
{7.38,114.01}
{7.55,115.06}
{7.55,128.70}
{6.95,129.20}
{7.39,129.08}
{6.93,129.17}
f1 (ppm)
f2 (ppm)

4.12. 1,1'-biphenyl-4-carbonitrile 14

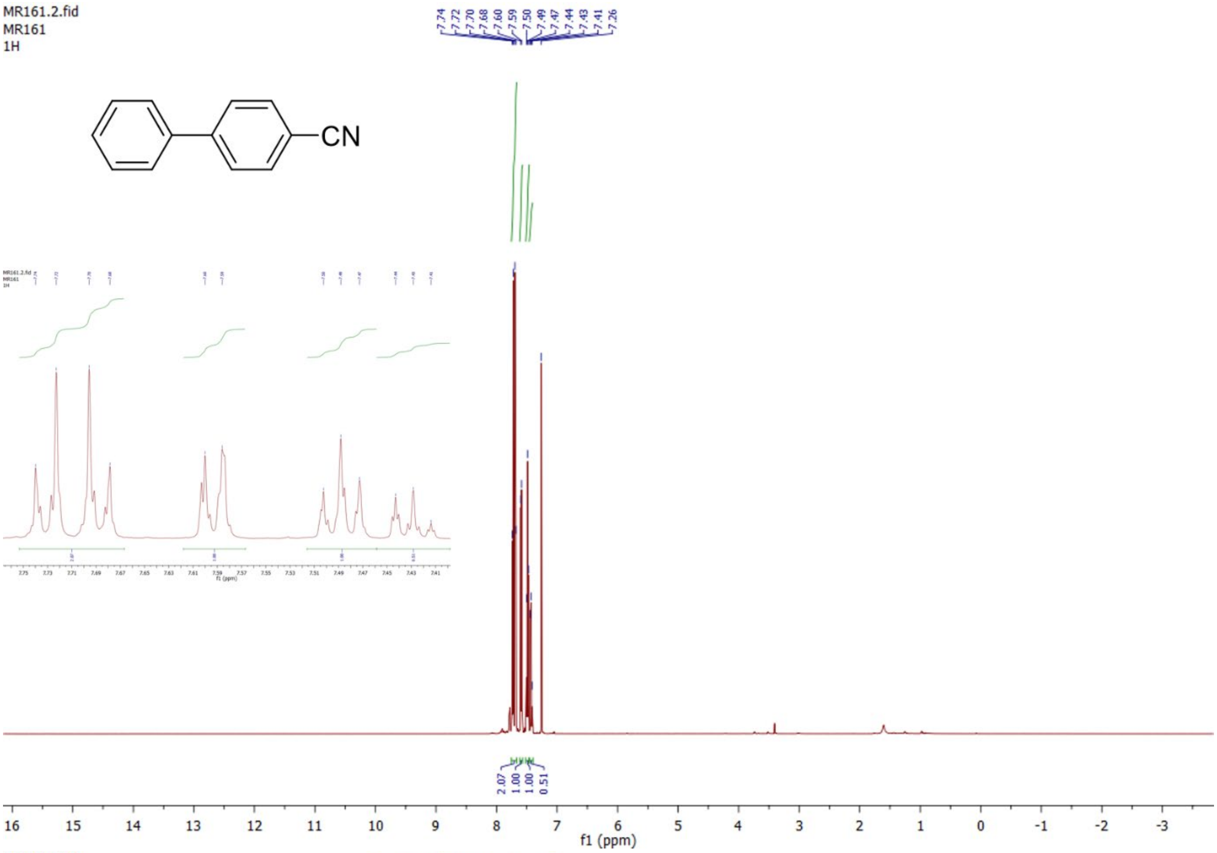

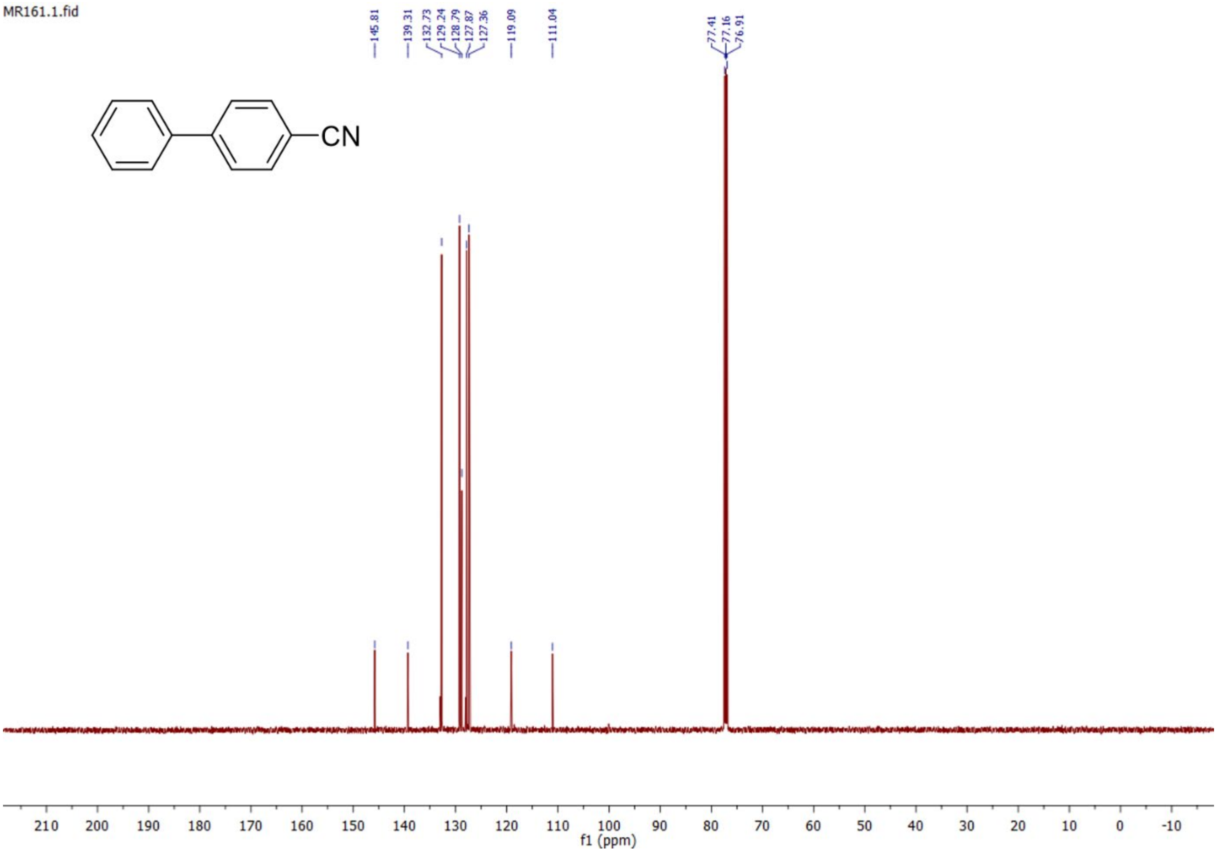

4.13. 4-nitro-1,1'-biphenyl 15

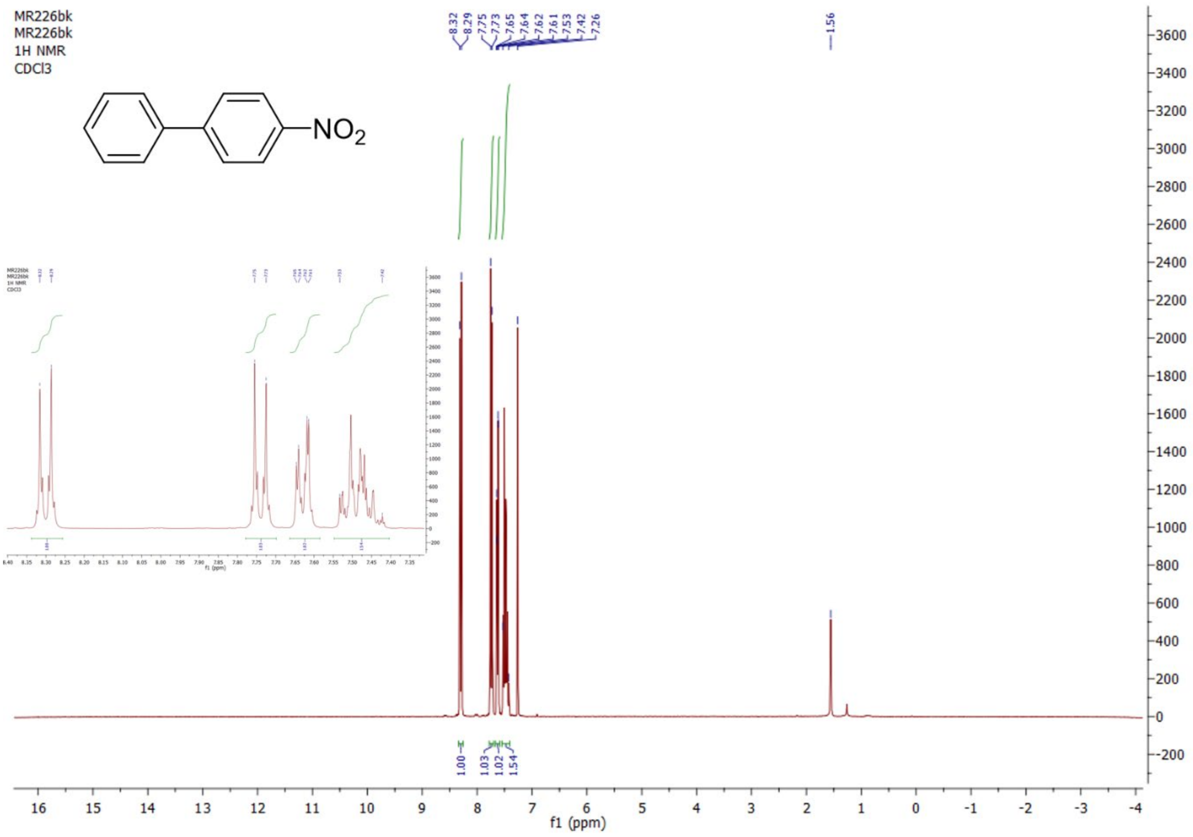

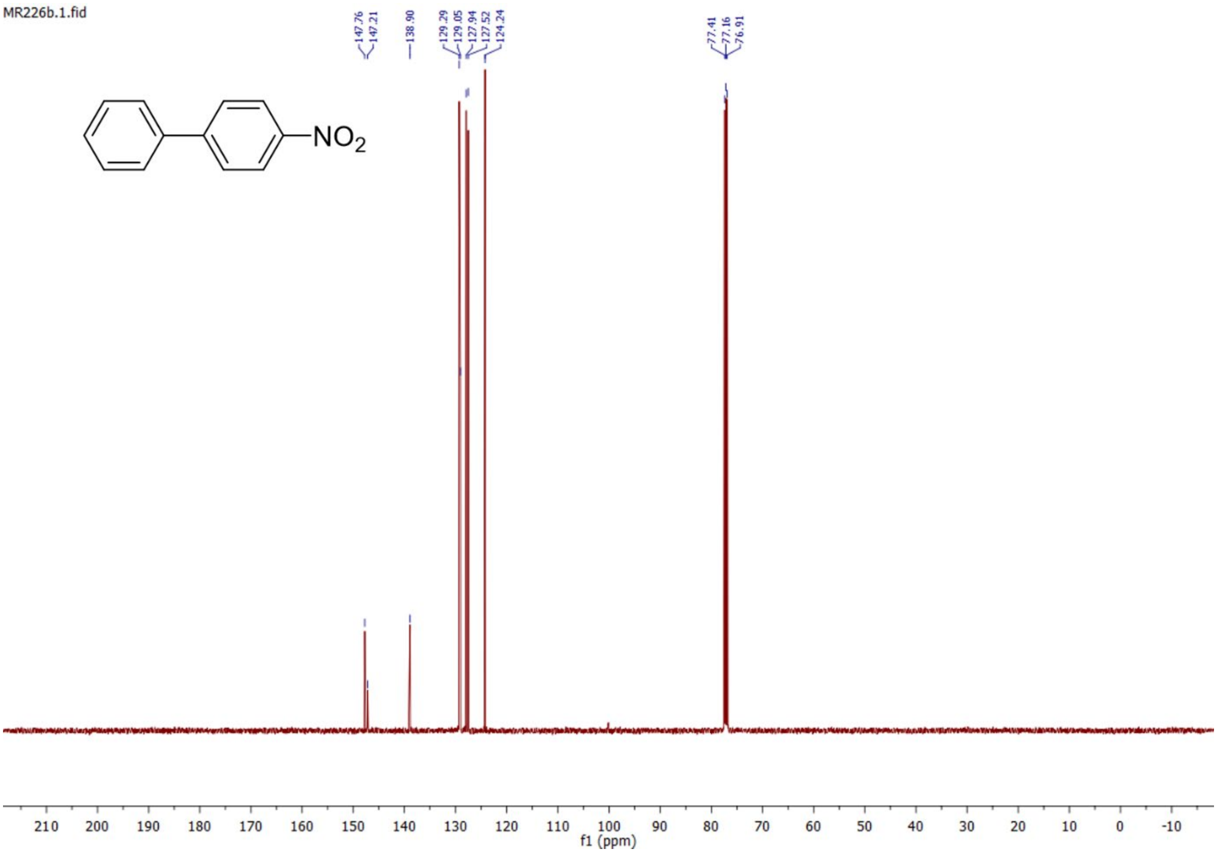

5. References

1 Koler, A.; Paljevac, M.; Cmager, N.; Iskra, J.; Kolar, M.; Krajnc, P. Poly(4-vinylpyridine) poly HIPEs as catalysts for cycloaddition click reaction. *Polymer*, **2017**, *126*, 402–407. https://doi.org/10.1016/j.polymer.2017.04.051

2 Zhang, G.; Luan, Y.; Han, X.; Wang, Y.; Wen, X.; Ding, C.; Gao, J. A palladium complex with functionalized β-cyclodextrin: a promising catalyst featuring recognition abilities for Suzuki–Miyaura coupling reactions in water. *Green Chem.*, **2013**, *15* (8), 2081–2085. https://doi.org/10.1039/C3GC40645H.
